# The switching role of β-adrenergic receptor signalling in cell survival or death decision of cardiomyocytes

**DOI:** 10.1038/ncomms6777

**Published:** 2014-12-17

**Authors:** Sung-Young Shin, Taeyong Kim, Ho-Sung Lee, Jun Hyuk Kang, Ji Young Lee, Kwang-Hyun Cho, Do Han Kim

**Affiliations:** 1 Department of Bio and Brain Engineering, Korea Advanced Institute of Science and Technology (KAIST), Daejeon 305-701, Korea; 2 School of Life Sciences and Systems Biology Research Center, Gwangju Institute of Science and Technology (GIST), Gwangju 500-712, Korea; 3 Graduate School of Medical Science and Engineering, KAIST, Daejeon 305-701, Korea

## Abstract

How cell fate (survival or death) is determined and whether such determination depends on the strength of stimulation has remained unclear. In this study, we discover that the cell fate of cardiomyocytes switches from survival to death with the increase of β-adrenergic receptor (β-AR) stimulation. Mathematical simulations combined with biochemical experimentation of β-AR signalling pathways show that the gradual increment of isoproterenol (a non-selective β_1_/β_2_-AR agonist) induces the switching response of Bcl-2 expression from the initial increase followed by a decrease below its basal level. The ERK1/2 and ICER-mediated feed-forward loop is the hidden design principle underlying such cell fate switching characteristics. Moreover, we find that β1-blocker treatment increases the survival effect of β-AR stimuli through the regulation of Bcl-2 expression leading to the resistance to cell death, providing new insight into the mechanism of therapeutic effects. Our systems analysis further suggests a novel potential therapeutic strategy for heart disease.

## Abstract (web summary)

The contribution of signal strength on cell fate decisions is often not reflected in signalling networks. By combining mathematical simulation and biochemical experiments in cultured adult cardiomyocytes, Shin *et al*. show that the concentration of a β-adrenergic receptor agonist affects the expression of Bcl-2, influencing the balance between cell survival and death.

## Graphical abstract

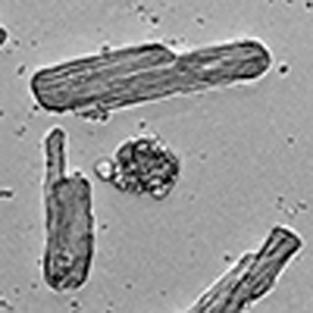

Recent systems biological studies have greatly advanced our understanding of key biological processes, such as cell fate decision (survival or death), in various cell lineages^1^,^2^,^3^,^4^,^5^. The mechanistic modelling of biological systems has enabled the identification of feedback and feed-forward loops in signalling networks^2^, which is essential for discovering the basic principles of cell fate decisions. According to Nakakuki *et al*.^5^, the distinct cell fate decision occurs through a coherent feed-forward loop that is interlinked with transcriptional negative feedback loops for epidermal growth factor and heregulin, whereas Chen *et al*.^2^ found that the homeostatic balance between precursor and differentiated cells is determined by the negative feedback mediated by Rasa2. In addition, Santos *et al*.^4^ found that different types of feedback loops work together for cell fate determination, depending on the type of growth factor stimulation.

In spite of advances in the mechanistic understanding of the cell fate decision through signalling networks, systematic studies to address the dependence of the cell fate decision on the strength of external stimuli for receptors such as the β-adrenergic receptor (β-AR) have not been conducted. The cardiomyocyte is an excellent model system for exploring these questions because cardiac cells are usually exposed to a wide concentration range of external stimuli under physiological conditions^6^,^7^,^8^,^9^. Moreover, multiple lines of experimental evidence have proposed that a prolonged β-AR stimulation can induce the cell death of cardiomyocytes^10^,^11^ and that the resulting reduction of cardiac contractility is related to the pathophysiology of heart failure^10^,^12^, suggesting the importance of β-AR signalling in controlling the progression of heart failure.

Previous studies have suggested that the β_1_-adrenergic receptor (β_1_-AR) transduces the ‘death’ signal via the cAMP-dependent signalling pathway, whereas the β_2_-AR transduces the ‘survival’ signal via the G_i_-coupled signalling pathway^13^,^14^. Such distinct roles for β_1_-AR and β_2_-AR were further supported by β_1_-/β_2_-AR double knockout mice^15^ and cardiac-specific overexpression of β_1_-AR and β_2_-AR^16^. Under physiological conditions, however, β-AR agonists such as isoproterenol (ISO) and catecholamine bind non-specifically to both types of receptors, which is puzzling because such non-specific binding should then convey both ‘survival’ and ‘death’ signals simultaneously^17^. In addition, β_1_-AR and β_2_-AR share some of their downstream signalling pathways, which are interlinked with each other through complicated feedback regulations^18^. Considering the structure of the β-AR signalling network and the non-specific binding characteristics of β-AR agonists, it is unclear how the β-AR subtypes are involved in the decision of cell survival or death. Rather, it is likely that the cell fate decision is the emergent outcome of collective interactions among various signalling molecules in the network.

Since Saucerman *et al*.^19^ developed a mathematical model that integrates β-AR signalling with excitation–contraction coupling, the β-AR model has been evolved for various research purposes^20^,^21^. All of these models, however, have addressed the functional roles of the β-AR signalling pathway that are associated with excitation–contraction coupling processes in cardiomyocytes without considering the cell fate decision. In the present study, we developed a novel and comprehensive mathematical model for the β-AR signalling network by integrating signal transduction, transcriptional regulation and Ca^2+^ regulation, all of which are closely associated with the cell fate decision in cardiomyocytes.

Through the model simulation and experimental validation, we found that a gradual increase of ISO induces a switching response of Bcl-2 and the resulting cell fate determination, which shows an initial increase of Bcl-2 and cell survival followed by a subsequent decrease of Bcl-2 and cell death. We further revealed that the ERK1/2 and ICER-mediated incoherent feed-forward loop is primarily responsible for such a differential response of Bcl-2 to the different range of ISO concentration. We also found that β_1_-blocker enhances the resistance of cardiomyocytes to cell death by expanding the survival range of the switching response curve of Bcl-2, providing a new insight into the mechanism of therapeutic effects and a novel potential therapeutic strategy.

## Results

### The mathematical model of the β-AR signalling network

**Figure 1:**
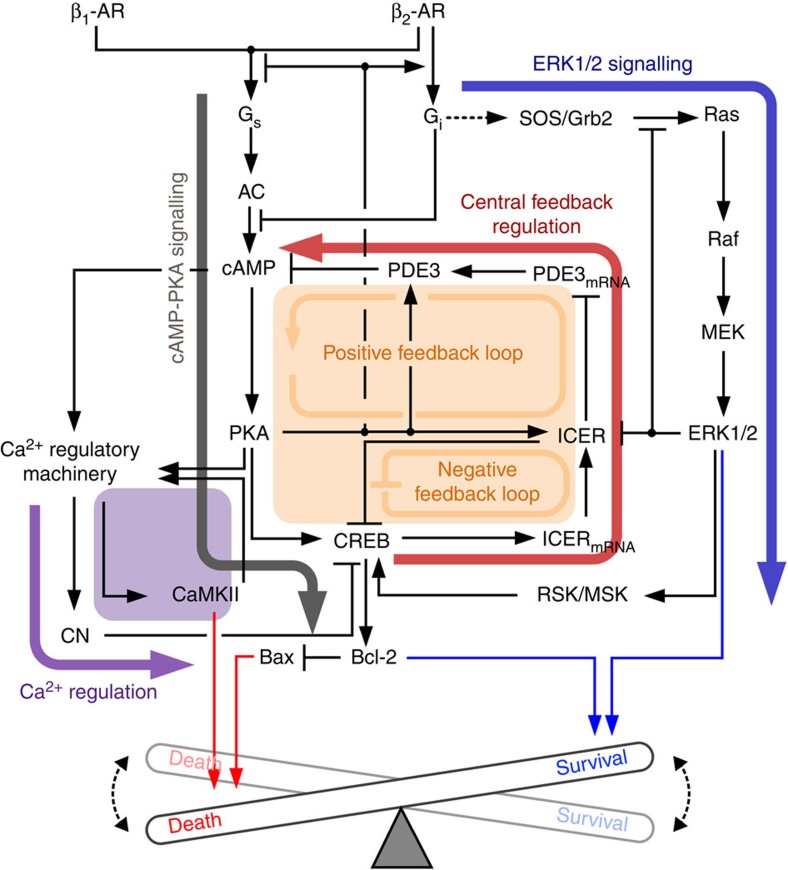
A schematic diagram for the β-AR signalling network. The β-AR signalling network comprises four modules: the cAMP-PKA signalling module (thick grey arrow), the central feedback regulatory module (thick red arrow and orange square), the ERK1/2 signalling module (thick blue arrow) and the Ca^2+^ regulatory module (thick purple arrow and purple square). β_1_-AR, β_1_-adrenergic receptor; β_2_-AR, β_2_-adrenergic receptor; G_s_, stimulatory G protein; G_i_, inhibitory G protein; AC, adenylyl cyclase; CN, calcineurin; ICER, inducible cAMP early repressor; RSK, stress-activated protein kinase; MSK, mitogen-activated protein kinase.

We have developed a novel and comprehensive mathematical model of the β-AR signalling network of cardiomyocytes to investigate the hidden cellular decision mechanism of cell survival or death. The reconstructed β-AR signalling network was based on the classical cAMP-protein kinase A (PKA) signalling pathway^19^,^22^, where all the interactions between signalling molecules were determined by extensive survey of available experimental data (see Supplementary Note 1 for details). The schematic diagram for the reconstructed β-AR signalling network has four major modules (cAMP-PKA signalling, the central feedback regulation, ERK1/2 signalling and Ca^2+^ regulation) (Fig. 1).

**Figure 2:**
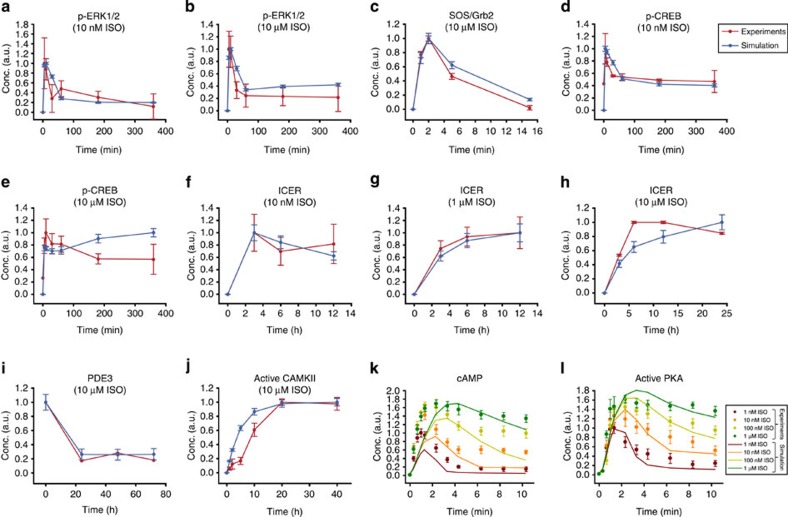
Estimation of kinetic parameter values for the β-AR signalling network model. The temporal profiles for signalling molecules in response to the indicated ISO concentrations were shown for the experimental results and the simulation results. The time courses of p-ERK1/2 (**a**,**b**), p-CREB (**d**,**e**) and ICER (**f**,**g**) were obtained from our experiments with primary adult rat cardiomyocytes. The time courses of the complex formation of SOS/Grb2 (ref. 67) (**c**), ICER protein^10^ (**h**), PDE3 protein^34^ (**i**), active CaMKII^68^ (**j**), cAMP^25^ (**k**), and PKA activity^25^ (**l**) were reproduced from the previous experimental data, where the cAMP levels and PKA activity were measured by real-time Förster resonance energy transfer-based live-cell imaging in the absence of PDE inhibitor. The kinetic parameters of the mathematical model were estimated from those experimental data. The experimental data represent mean±s.e.m. for at least three independent experiments; representative blot images and the time course of qRT–PCR results can be found in Supplementary Fig. 1. The simulation data represent mean±s.e.m. (or mean value) for the repetitive simulations (*n*=30) over up to 30% random variation of parameter values.

Our model comprises 32 state variables and 105 kinetic parameters (see Supplementary Tables 1–4). The total protein concentrations (constant values) were estimated from our RNA-seq data^23^ (see Supplementary Table 5 for details). To estimate the kinetic parameter values, we used time course data for signalling molecules in the network, such as ERK1/2, SOS/Grb2, CREB, ICER, CaMKII, PDE3, cAMP and PKA. As shown in Fig. 2, the simulation data fit well to the experimental data (Fig. 2a–l).

### Isoproterenol induces dose-dependent cell survival or death

**Figure 3:**
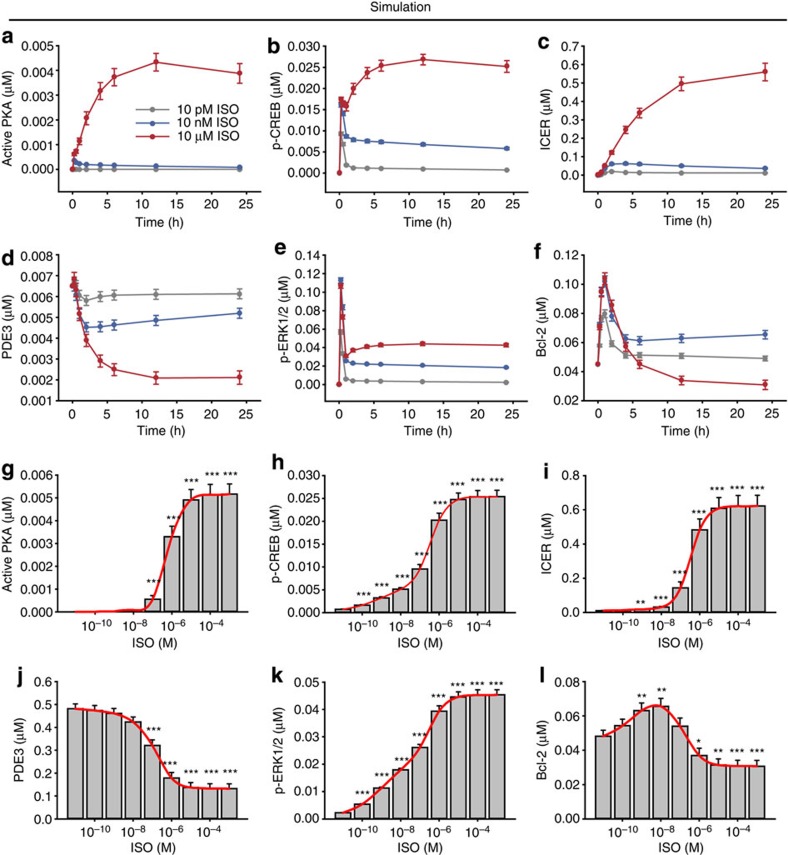
ISO induces the switching response of Bcl-2. Time-dependent PKA activation (**a**), CREB phosphorylation (**b**), ICER expression (**c**), PDE3 expression (**d**), ERK1/2 phosphorylation (**e**) and Bcl-2 expression (**f**) in response to three different concentrations of ISO: 10 pM (grey line), 10 nM (blue line) and 10 μM (red line). The time courses of these signalling molecules were quite distinct depending on the ISO concentration. The dose–response profile of PKA activation (**g**), CREB phosphorylation (**h**), ICER expression (**i**), PDE3 expression (**j**), ERK1/2 phosphorylation (**k**) and Bcl-2 expression (**l**) for the indicated ISO concentration range are also shown. Each dose response (**g**–**l**) was observed at 24 h after the ISO stimulation. Active PKA, p-CREB, ICER, PDE3 and p-ERK1/2 showed sigmoidal dose–response profiles, and Bcl-2 expression increased at the nanomolar concentration range; however, it then decreased to further below its basal level at a micromolar concentration range. The data represent means±s.e.m. for the repetitive simulations (*n*=100) over up to 20% random variation of parameter values. *P<0.05; **P<0.01; ***P<0.001 compared with control group; Student’s *t*-test.

It has been suggested that the survival or death of cardiomyocytes is determined by the receptor type^13^,^14^. For example, β_1_- and β_2_-AR mediate the death and survival signal, respectively. On the other hand, Henaff *et al*.^24^ reported that a specific concentration range (0.01–1 μM) of epinephrine protects cardiomyocytes from apoptosis. To examine the hypothesis that the cell fate of cardiomyocytes depends on the β-agonist concentration, we simulated the cellular responses to β-AR signalling by using three representative ISO concentrations: 10 pM, 10 nM and 10 μM. The simulation results showed contrasting time courses of signalling with respect to different ISO concentrations (Fig. 3a–f; see also Supplementary Fig. 2 for the expression patterns of all the signalling components). Active PKA, p-CREB, ICER and PDE3 showed transient response profiles at 10 pM and 10 nM, whereas sustained response profiles appeared at 10 μM. Note that p-ERK1/2 showed a transient response profile at 10 pM but showed sustained response profiles at 10 nM and 10 μM. All of these time course profiles are compatible with the previous findings that showed a transient response to lower ISO stimulation and a sustained response to higher ISO stimulation 25 (Fig. 3a–e). Interestingly, Bcl-2 exhibited distinct response profiles (Fig. 3f); it showed transient and sustained responses for 10 pM and 10 nM, respectively, but it showed an initial increase followed by a subsequent decrease to below its basal level (~50%) on stimulation with 10 μM ISO.

For further investigation of cellular responses over a broad concentration range of ISO, we simulated dose–response profiles for each signalling molecule at ISO concentrations that ranged from 10^−12^ to 10^−3^ M. The active PKA, phospho-CREB, phospho-ERK1/2 and ICER increased monotonically along with the increase of ISO concentration, whereas PDE3 decreased since ICER transcriptionally represses its induction (Fig. 3g–k). However, Bcl-2 exhibited a quite distinct ‘switching response’ profile, with an initial increase at the nanomolar concentration range of ISO followed by a further decrease to below its basal level (~50%) in a micromolar concentration range (Fig. 3l). Bcl-2 is known to inhibit mitochondrial apoptosis and necrosis^26^,^27^, and the survival effect of Bcl-2 is largely supported by other findings such as the reduction of ATP consumption and the inhibition of autophagy^28^,^29^,^30^. Therefore, the increase of Bcl-2 can protect cardiomyocytes from cell death, whereas its decrease can promote cell death^31^,^32^,^33^. Together our simulation results show that the survival or death of cardiomyocytes might depend on the stimulation strength of β-AR, given by ISO concentration in our study, through the regulation of the expression level of Bcl-2.

**Figure 4:**
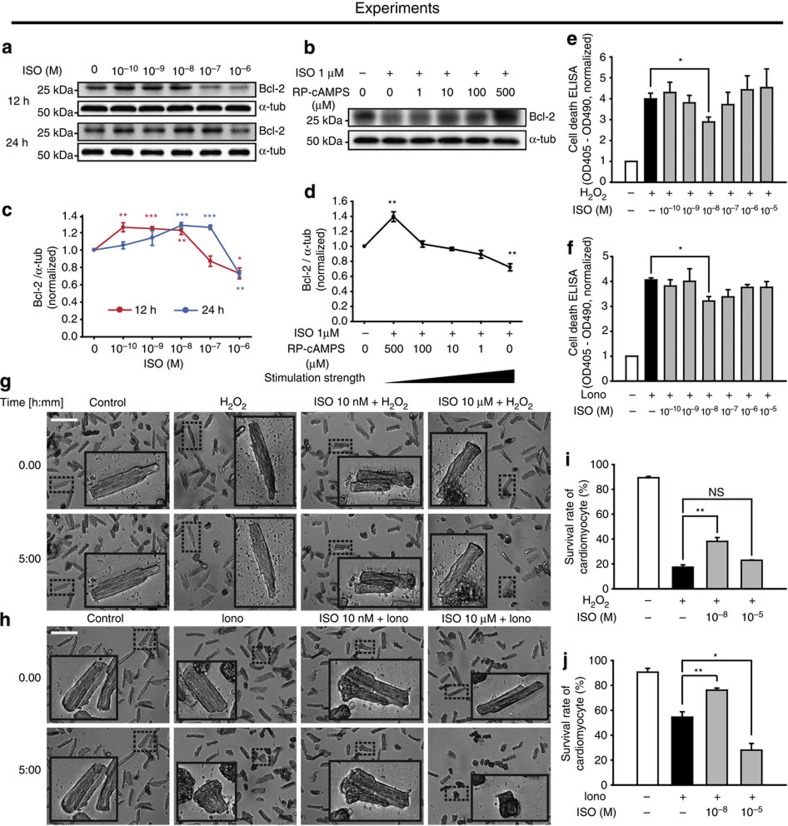
Switching response of Bcl-2 to β-AR stimulation in cardiomyocytes. (**a**–**d**) Switching response profiles of Bcl-2 were observed in cardiomyocytes when we incubated them with the indicated concentrations of ISO for 12 or 24 h (**a**) or preincubated them with the indicated concentrations of a PKA inhibitor (RP-cAMPs) for 2 h before incubation with 1 μM ISO for 12 h (**b**). Representative Bcl-2 and α-tubulin (loading control) immunoblots (**a**,**b**) and the semi-quantified data (**c**,**d**) are shown. Note that the concentrations of RP-cAMP were rearranged in the order of increased signalling flux through the cAMP-PKA signalling module (**d**). The data represent means±s.e.m., *n*=3 biological and technical replicates (independent culture preparations). (**e**–**j**) Cardiomyocytes preincubated with the indicated concentrations of ISO were subjected to H_2_O_2_ (100 μM) or ionomycin (3.5 μM) treatment. Cell death was measured by ELISA (**e**,**f**). The survival rate was assessed by live-cell imaging (**g**,**h**; Supplementary Movies 3–9), and the data were summarized (**i**,**j**), where the insets are magnifications of the dash-outlined area (**g**,**h**). Scale bar, 100 μm. The data represent means±s.d. pooled from three biological and technical replicates (independent culture preparations) (**e**,**f**) or means±s.e.m. pooled from three biological and technical replicates (independent culture preparations) (**i**,**j**). *P<0.05; **P<0.01; ***P<0.001; NS, not significant compared with their control groups; Student’s *t*-test. Uncropped western blots are shown in Supplementary Fig. 14.

To verify this possibility, we stimulated isolated adult cardiomyocytes over a broad concentration range of ISO (10^−10^–10^−6^ M) for 12 or 24 h, respectively, and we observed the resulting Bcl-2 expression (Fig. 4a,c). Bcl-2 expression increased significantly at lower concentrations (10^−10^–10^−8^ M for 12 h and 10^−8^–10^−7^ M for 24 h) but decreased at higher concentrations (10^−6^ M), as predicted by the simulation data (Fig. 3l). Note that only 10^−8^ M showed the significant increase of Bcl-2 both at 12- and 24-h time points.

To examine whether the cell fate of cardiomyocytes is determined by a specific isoform of β-AR, the signal flux through the cAMP-PKA signalling module was controlled by using a broad concentration range of RP-cAMPS (a PKA inhibitor) before treatment with 1 μM ISO. If the cell fate is determined at the level of receptors, the downstream signalling flux control would not have any conclusive effect on the cell fate. However, the result showed a clear switching response curve for Bcl-2 (Fig. 4b,d) as well as for the survival rate of cardiomyocytes (Supplementary Fig. 3 and Supplementary Movie 1). Note that the results for p-phospholamban at S16 (a specific substrate for PKA) and p-CREB showed a linearly increasing pattern, but not a switching response (Supplementary Fig. 4). Together these results suggest that it is the complex interaction of downstream signalling molecules and not the specific type of receptors that determines the cell fate of cardiomyocytes in case the binding ligand is non-specific to the receptors. Given that Bcl-2 is a strong anti-apoptotic (or prosurvival) factor in cardiomyocytes^10^,^34^,^35^, ISO in the nanomolar concentration range should promote the survival of cardiomyocytes. To test this hypothesis, cardiomyocytes pretreated with 0.1–10 μM ISO for 12 h were treated with a strong nonspecific apoptosis inducer, that is, H_2_O_2_ or ionomycin. Cell death measured by enzyme-linked immunosorbent assay (ELISA) decreased only at 10 nM ISO (Fig. 4e,f). This ISO concentration of the survival effect is in accord with the concentration that showed the peak amount of Bcl-2 regardless of the stimulation duration (Fig. 4c). To validate whether the changes in Bcl-2 expression levels cause cAMP-mediated apoptosis, we conducted quantitative reverse transcription-PCR (qRT–PCR) analysis of pro- and anti-apoptotic family members that are known to be regulated by cAMP (for example, Bim and IAP family members). The expression levels of the pro-apoptotic proteins Xiap, cIAP2 and Bim did not show any significant changes in response to ISO stimulation even at 1 μM. The anti-apoptotic protein cIAP1 was transiently increased for the initial 12 h and then decreased to its basal level; however, this change does not seem to be relevant to the induction of cell death by ISO at the higher concentration since the upregulation of such anti-apoptotic protein may not cause apoptosis (Supplementary Fig. 5). Taken together, the previous evidence^10^,^34^,^35^ and the present results suggest that Bcl-2 is an important mediator that determines the survival or death of cardiomyocytes depending on the concentration of ISO.

To validate the survival effect of ISO, we counted the number of live cardiomyocytes that contained intact sarcomeric structures by time-lapse live-cell imaging (see Supplementary Movies 2–8) because disruption of the sarcomeric structure is the key morphological feature for apoptotic cardiomyocytes (see Supplementary Movies 9 and 10)^31^,^36^. The survival rate of cardiomyocytes increased substantially only at 10 nM (Fig. 4g–j), which is consistent with the ELISA results. Note that the contrasting cell fate determination becomes evident for any longer duration than 6 h of stimulation irrespective of the ISO level, while the cell fate determination does not change by the duration of the stimuli (Supplementary Fig. 6). Collectively, these results suggest that the strength of β-AR stimulation determines cell fate in cardiomyocytes.

As shown previously, we also quantified the relative amount of apoptosis by measuring the expression ratio of Bax (a pro-apoptotic counterpart of Bcl-2) to Bcl-2 (refs 37, 38). The expression of Bax was almost constant over the indicated concentration range of ISO (Supplementary Fig. 7), but Bcl-2 showed a switching response curve (Fig. 4a,c), as predicted from our model simulation (Fig. 3l).

### Identification of the core circuit for cell fate switching

**Figure 5:**
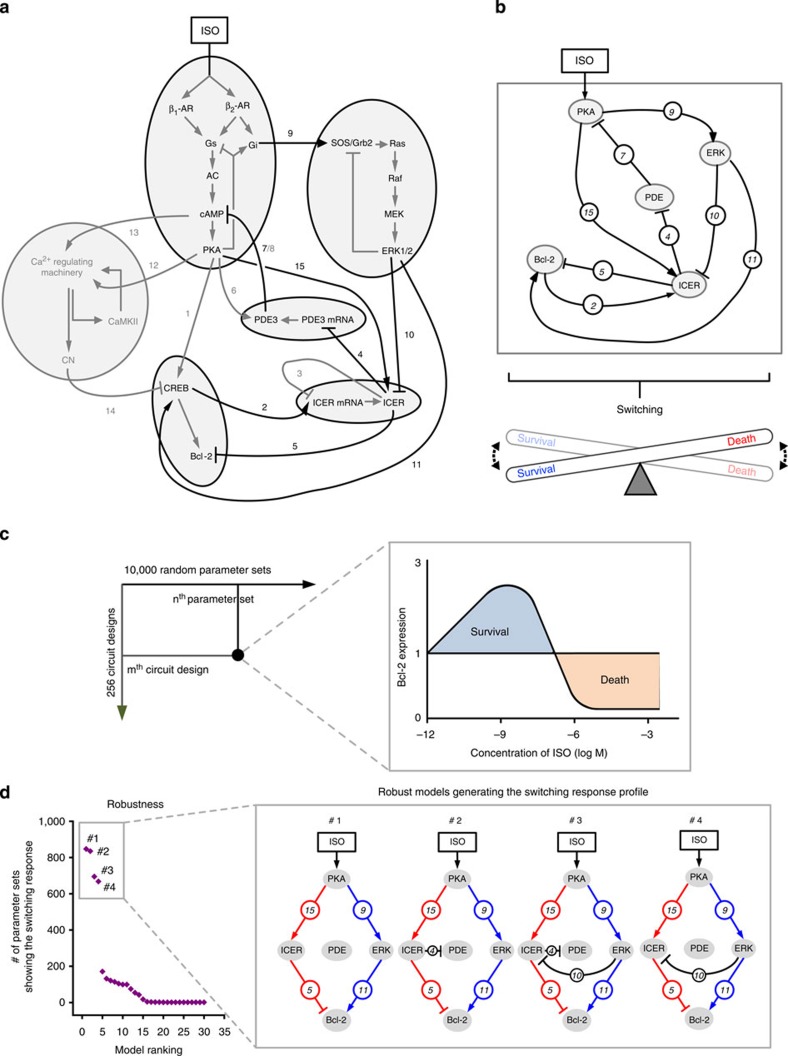
Identification of the core regulatory circuit that robustly generates the switching response profile of Bcl-2. (**a**) A detailed β-AR signalling network that implements the switching response of the Bcl-2 expression. The grey-shaped circles indicate clustered signalling components. (**b**) A simplified β-AR signalling network. The eight links are essential for the generation of the switching response of Bcl-2. The circled numbers indicate the indexes of the regulatory links that are same as in **a**. (**c**) Identification of models that can robustly generate the switching response profile of Bcl-2 for each combination of circuit design (m^th^) and parameter set (n^th^). Inset: the identification is constrained to the switching response of Bcl-2 to the gradual increase of ISO. (**d**) The four most robust models that can produce the switching response of Bcl-2, in which ERK and ICER-mediated incoherent feed-forward loop is commonly included.

To identify the core regulatory circuit of the β-AR signalling network that is responsible for the Bcl-2 switching response profile, we applied the coarse-graining method that can reduce the complexity of a network while keeping its essential regulatory features^39^,^40^. Using this method, we clustered all the signalling components into a set of functional units (Fig. 5a). For instance, β-ARs, G_s_, G_i_, cAMP and PKA were combined into one functional unit ‘PKA’, and SOS/Grb2, Ras, Raf, MEK and ERK1/2 were grouped into another unit ‘ERK’. Similarly, the transcriptional processes of ICER and PDE3 were combined into ‘ICER’ and ‘PDE’, respectively. The linear signalling cascades consisting of CREB and Bcl-2 were combined into ‘Bcl-2’. Finally, all Ca^2+^ related components were combined together as one functional unit. As a result, the β-adrenergic signalling network was reduced to a coarse-grained network composed of six functional units and 15 regulatory links (Fig. 5a). In the next, to identify essential regulatory links that are responsible for the switching response profile of Bcl-2, we simulated all possible combinations of the perturbed regulatory circuits, that is, a total of 2^15^ (=32,768) rewired networks in which each link is either connected or disconnected and, as a result, we found the eight essential regulatory links (that is, , , , , , , and ) that are primarily responsible for the switching response of Bcl-2 (Fig. 5b).

To further investigate the hidden design principle underlying the switching response, we considered the simplified model composed of five nodes (that is, PKA, PDE, ERK, ICER and Bcl-2) corresponding to the clustered functional units and eight essential regulatory links among them (see Supplementary Note 2 for the simplified ordinary differential equation (ODE) model; Fig. 5b). Then, we produced 2^8^ (=256) network models by considering all possible rewired structures obtained by perturbing each of the eight essential regulatory links of the simplified model and simulated each rewired network model with 10,000 random parameter sets (Fig. 5c). Note that such different parameter sets can represent various cellular contexts and different cell types. From simulation analysis, we found that only a small set (30 out of 256) of the network models can robustly generate the Bcl-2 switching response profile against such random parameter variations and that the four most robust regulatory circuits commonly include the ERK and ICER-mediated incoherent feed-forward loop (Fig. 5d). Here the robustness of a regulatory circuit was measured by the number of parameter sets (out of 10,000 sets) that allow the model to generate the Bcl-2 switching response profile 40. Together these results suggest that the core regulatory circuit composed of the ERK and ICER-mediated incoherent feed-forward loop plays a crucial role in robustly generating the Bcl-2 switching response of cardiomyocytes and therefore it forms the hidden design principle underlying the switching response of Bcl-2.

**Figure 6:**
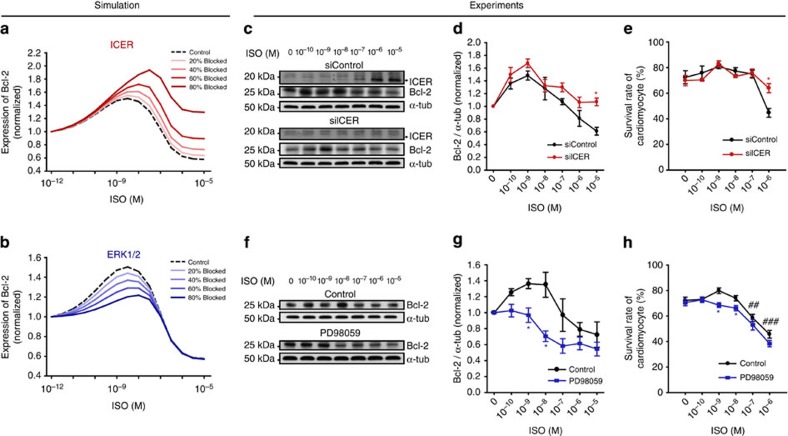
ISO concentration-dependent Bcl-2 expression for a different perturbation level of each essential regulatory link. (**a**) Blockade of ICER-mediated link significantly increased Bcl-2 expression only at or above the micromolar concentration range of ISO. (**b**) Blockade of the ERK1/2-mediated link decreased Bcl-2 expression at 10^−10^–10^−7^ M ISO, where the dashed line denotes control and the solid line denotes the perturbed condition. The simulation of the dose–response profiles was observed at 12 h after ISO stimulation. (**c**–**e**) Control siRNA (siControl) or ICER siRNA (siICER) transfected cardiomyocytes were stimulated with the indicated concentrations of ISO. (**c**) Representative immunoblots showing Bcl-2 expression at the indicated ISO concentration determined in the presence of siControl or siICER. (**d**) Plots of Bcl-2 protein expression shown in **c** versus ISO dose. Data represent means±s.e.m., *n*=3 biological and technical replicates (independent culture preparations). (**e**) The survival rate was assessed by live-cell imaging. Data represent means±s.e.m., *n*=3 biological and technical replicates (independent culture preparations). (**f**–**h**) Cardiomyocytes were stimulated with the indicated concentrations of ISO in the presence or absence of PD98059 (f) Representative immunoblots showing Bcl-2 expression at the indicated ISO concentration determined in the presence or absence of PD98059. (**g**) Plots of Bcl-2 protein expression shown in **f** versus ISO dose. Data represent means±s.e.m., *n*=3 biological and technical replicates (independent culture preparations). (**h**) The survival rate was assessed by live-cell imaging. Data represent means±s.e.m., *n*=6 biological and technical replicates (independent culture preparations). ^##^*P*<0.01; ^###^*P*<0.001 compared to non-treated control group; *P<0.05 compared with no drug-treated and the same concentration of ISO-treated group; Student’s *t*-test. Uncropped western blots are shown in Supplementary Fig. 14.

To extend our investigation of the core regulatory circuit in producing the switching response profile of Bcl-2, we gradually inhibited the incoherent feed-forward loop by 20 to 80%. The blockade of ICER-mediated negative regulation ( in Fig. 5d) remarkably increased Bcl-2 expression only at a micromolar concentration range of ISO (Fig. 6a) whereas the blockade of ERK1/2-mediated positive regulation ( in Fig. 5d) decreased Bcl-2 expression mostly at a nanomolar range of ISO (Fig. 6b). For comparison, we further simulated the switching response profile of Bcl-2 by inhibiting the cAMP-PKA signalling and found that the switching profile of Bcl-2 disappears after the PKA inhibition (Supplementary Fig. 8). Therefore, the ERK1/2 and ICER-mediated incoherent feed-forward loop differentially regulates the Bcl-2 response depending on the concentration range of ISO, which enables the cell to choose a different cell fate depending on the strength of the external stimulus.

To validate the simulation results, we biochemically blocked the ERK1/2- and ICER-mediated feed-forward loop alternately and measured the expression of Bcl-2. First, small interfering RNA (siRNA) was used to block the ICER-mediated negative regulation of the incoherent feed-forward loop, which was expected to inhibit *de novo* synthesis of the ICER protein efficiently as shown previously^41^. siRNA transfection into adult cardiomyocytes was successful (Supplementary Fig. 9). ICER siRNA (siICER) completely blocked the induction of ICER protein expression at ISO stimulation for 12 h (Fig. 6c). Three hours after siRNA transfection, cardiomyocytes were stimulated with various concentrations of ISO for 12 h. Then we measured the Bcl-2 protein level by western blotting (Fig. 6c). siICER increased Bcl-2 protein significantly at the micromolar concentration range of ISO compared with siControl. However, it did not affect the Bcl-2 protein level at the nanomolar concentration range, concordant with our prediction (Fig. 6a). Moreover, the concentration-specific effects of siICER were also observed in the survival rate. The survival rate at the nanomolar concentration range of ISO was not affected by siICER, but the survival rate at the micromolar concentration range was significantly decreased (Fig. 6e and Supplementary Movie 11).

In the next, we treated the cells with PD98059 (a MEK inhibitor) to block the ERK1/2-mediated positive regulation of the incoherent feed-forward loop. To assess the effect of PD98059, we measured the protein level of Bcl-2. Semi-quantitative analysis by western blotting showed that the expression of Bcl-2 decreased considerably at the nanomolar concentration range of ISO, whereas the decrement was relatively small at the micromolar concentration range of ISO (Fig. 6f,g), and the measurement of the survival rate showed similar effects (Fig. 6h and Supplementary Movies 12 and 13), which is in accord with the model prediction (Fig. 6b).

In addition, we investigated the functional role of the ERK1/2-mediated positive regulation of the incoherent feed-forward loop using the RSK inhibitor (BRD7389) since the RSK phosphorylation of CREB is an important mechanism by which ERK1/2 promotes cell survival. The RSK inhibition showed the same behaviour; the significant decrease of Bcl-2 at 1–10 nM, compared to the non-treatment group, which is similar to the MEK inhibition (Supplementary Fig. 10). Taken together, the results suggest that the distinct function of ERK and ICER-mediated incoherent feed-forward loop enables the switching of cellular decision from survival to death depending on the level of ISO concentration.

### β_1_-blocker enhances the tolerance of cardiomyocytes to death

β-blockers are widely used as effective therapeutic agents for heart failure patients^42^. Some evidence has shown a positive correlation between treatment with β-blockers and the rate of cell survival^43^,^44^. For instance, the β-blocker reduces oxidative stress^45^,^46^, ameliorates myocardial creatine dysregulation^47^, improves the biological function of the failing heart muscle^48^ and relieves endoplasmic reticulum-stress and endoplasmic reticulum-mediated apoptosis^49^. Despite its extensive clinical use and animal experiments, the underlying mechanism by which β-blockers increase survival in heart failure patients remains unclear.

**Figure 7:**
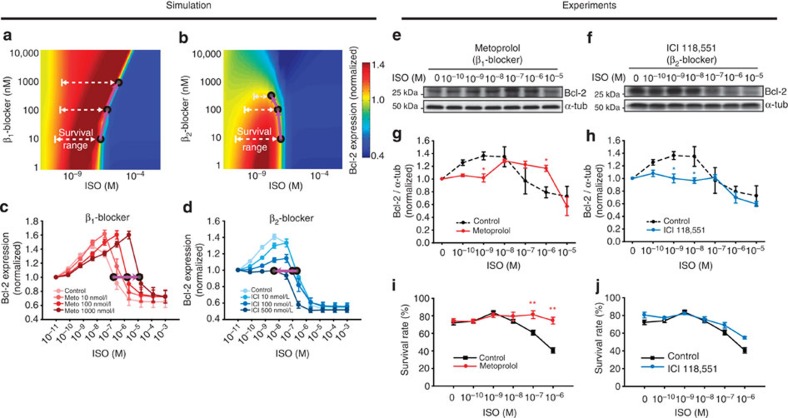
β_1_-blockers increase the tolerance of cardiomyocytes to cell death by expanding the survival range of the switching response profile of Bcl-2. (**a**,**b**) Heat maps of Bcl-2 expression in response to treatment with ISO and β_1_-blocker (**a**) or ISO and β_2_-blocker (**b**). The survival range was increased by the β_1_-blocker, whereas it was decreased by the β_2_-blocker. (**c**,**d**) Simulation curves for Bcl-2 expression versus ISO dose in the presence of different concentrations of β_1_-blocker (**c**) or β_2_-blocker (**d**). Note that the bell-shaped curves were expanded toward a high ISO concentration with an increased β_1_-blocker concentration, whereas the opposite pattern was observed for the β_2_-blocker. The simulation of the dose–response profiles was observed at 24 h after ISO stimulation. The data represent mean±s.e.m. for the repetitive simulations (*n*=20) over up to 20% random variation of parameter values. (**e**,**f**) Representative immunoblots showing the ISO concentration-response effects for Bcl-2 expression in the presence of metoprolol or ICI 118,551. (**g**,**h**) The line graphs depict the semi-quantification of the immunoblots shown in **e**,**f**. The dashed lines represent the concentration-response profile of Bcl-2 expression with ISO alone (that is, the control data taken from Fig. 6i). (**i**,**j**) The survival rate was assessed by live-cell imaging in the presence of metoprolol or ICI 118,551. Cell death induced by higher concentration (10^−7^–10^−6^ M) of ISO is significantly reduced by metoprolol. Data represent means±s.e.m. pooled from more than three biological and technical replicates (independent culture preparations). *P<0.05; **P<0.01 with Student’s *t*-test. Uncropped western blots are shown in Supplementary Fig. 14.

Our experimental results and mathematical simulation suggested that ISO induces the switching response of Bcl-2, and the fate of cardiomyocytes depends on the concentration of ISO (Figs 3, 4, 5, 6). Thus, we further simulated the concentration-response profiles of Bcl-2 to ISO for a wide concentration range (10^−11^ to 10^−4^ M) of the β_1_-blocker or β_2_-blocker. The β_1_-blocker broadened the ISO-mediated survival range due to the increased Bcl-2 expression (Fig. 7a). The expanded range of survival was also obvious in the switching response profile of Bcl-2 (Fig. 7c). In contrast, the β_2_-blocker narrowed down the survival range (Fig. 7b) by shifting the switching response profile of Bcl-2 to the left (Fig. 7d).

To validate the model prediction, we preincubated cardiomyocytes with 10 μM metoprolol (a selective β_1_-blocker) or 500 nM ICI 118,551 (a selective β_2_-blocker), and then treated them with the indicated concentrations of ISO. Metoprolol shifted the switching response profile of Bcl-2 to the right (Fig. 7e,g) and significantly enhanced the cell survival rate at higher concentrations (10^−7^–10^−6^ M; Fig. 7i, Supplementary Movies 14 and 15) of ISO, which is concordant with the model prediction shown in Fig. 7c. ICI 118,551 significantly suppressed the Bcl-2 expression at lower concentrations of ISO and slightly shifted the response profile to the left, which agrees with the model prediction shown in Fig. 7d. Taken together, these results suggest that β_1_-blockers increase the tolerance of cardiomyocytes to cell death by expanding the survival range of the switching response curve of Bcl-2.

## Discussion

The β-ARs are a class of G protein-coupled receptors that are the targets of catecholamine (for example, norepinephrine and epinephrine) and their subtypes, β_1_- and β_2_-ARs are thought to mediate the death and survival signals, respectively. However, it has been difficult to understand the net effects of β-agonists on cell fate decisions through traditional biological approaches since both types of β-ARs are the targets of catecholamine. This issue is particularly important from the clinical point of view since heart failure patients are known to have an increased sympathetic activity resulting in prolonged β-AR stimulation, which eventually leads to the death of cardiomyocytes. Since the progressive loss of cardiomyocytes is considered to play a major role in heart failure, controlling the cell death of cardiomyocytes caused by β-AR stimulation is one of the principal challenges to cure the disease. In the present study, we attempted to identify the mechanistic principle underlying the cell fate decision process through a systems biological approach^50^,^51^,^52^. The proposed approach can be useful for developing new therapeutic strategies since the available data from the conventional reductive approaches are often partial, heterogeneous and sometimes controversial, and thereby it still remained mostly unclear how the signalling molecules coordinate together and make the cell fate decision.

Our findings obtained by developing a comprehensive mechanistic model (Figs 1 and 2) led to the following conclusions: (1) The cell fate of cardiomyocytes is switched from survival to death depending on the stimulation strength of β-ARs, as a result of changes in Bcl-2 expression (Figs 3 and 4); (2) the ERK1/2 pathway at a low concentration range of ISO positively regulates Bcl-2 induction and thus cell survival ensues, while the PKA-CREB-ICER pathway activated at a high concentration range negatively regulates Bcl-2 induction and promotes cell death (Figs 5 and 6); (3) Metoprolol (a β1-blocker) shifts the switching response profile of Bcl-2 towards a high ISO concentration range and therefore significantly enhances the cell survival rate at a high concentration of ISO whereas ICI 118,551 (a β2-blocker) slightly shifts the response profiles towards a low ISO concentration range, providing a critical basis for the development of a better drug (Fig. 7).

Although the ISO concentration in the majority of previous experiments was in the range of 0.1–10 μM where cell death is usually observed^10^,^15^,^34^,^53^,^54^, ISO has been widely used for the treatment of bradycardia and heart block^55^. Given the contrasting effects of ISO that were shown in our study, the adverse effects of ISO might not be necessarily inherent to the drug itself, but depend on the concentration range of its use. Therefore, a large-scale clinical investigation is required to confirm the differential effects of ISO at low and high concentrations and to establish new guidelines for its clinical use.

Although the β-AR system is an essential compensatory mechanism that increases cardiac output under physiological conditions, the prolonged stimulation of β-ARs has been known to induce apoptosis in cardiomyocytes^10^,^12^. The survival effect of β-AR stimulation at low agonist concentrations discovered in the present study enables the β-AR system to regulate the cardiac function with substantial stability below the threshold agonist concentration. In the case of heart failure patients who have highly increased norepinephrine content, β_1_-blocker treatment may raise this threshold, thereby protecting the cardiomyocytes from β-AR-induced apoptosis (Fig. 7).

In the previous study, Ding *et al*.^35^ found that PDE3A is the key mediator of the ICER feedback loop, and it regulates Bcl-2 expression directly. They also showed that the expression of PDE3A was decreased significantly in the failing heart and that the overexpression of PDE3A in cardiomyocytes prevented cell death. These observations are consistent with our model prediction and experimental results, as ICER-mediated PDE3 regulation is included in the essential regulatory links that we found (Fig. 5b) and the biochemical blockade of ICER-mediated link markedly increased Bcl-2 expression at micromolar ISO concentrations (Fig. 6d,e), resulting in a significant reduction of the cell death of cardiomyocytes (Fig. 6g). Thus, interruption of the PDE3A-ICER link may be a novel therapeutic strategy.

As shown in Fig. 7f,h, the β_2_-blocker narrowed down the survival range of the switching response profile of Bcl-2 and decreased the expression of Bcl-2 at low concentrations of ISO. This undesirable effect of the β_2_-blocker can be explained if the β_2_-blocker inhibits the coupling of β_2_-AR to G_i_, which would prevent the activation of the ERK1/2 signalling module (Fig. 1) and subsequently decrease the expression level of Bcl-2. Hence, the use of β_2_-blockers would be toxic to the cells, but the use of β_2_-agonists may be therapeutically beneficial. Our findings are supported by a recent study that demonstrated a cardioprotective therapy using β_2_-AR agonists and β_1_-AR blockers in a rat dilated cardiomyopathy model^56^,^57^.

To further investigate the therapeutic targets for heart failure, we performed sensitivity analyses (local sensitivity analysis (LSA) and global sensitivity analysis (GSA)) of the network model by examining the influence of parameter perturbations on Bcl-2 expression (see Methods for details). For this purpose, we selected a set of kinetic parameters that are directly related to the potential therapeutic targets as summarized in Supplementary Table 6. The LSA and GSA sensitivity scores show that the model parameters related to the AC, PDE3 and ICER have significant effects on the Bcl-2 expression (Supplementary Fig. 11a,b). All of these parameters are associated with the eight essential links which are primarily responsible for the Bcl-2 switching response of cardiomyocytes (Fig. 5a,b; the result of additional sensitivity analysis on the Bcl-2 switching response profile is shown in Supplementary Fig. 12 and Supplementary Note 3). To evaluate the therapeutic effects of these three potential targets, we further simulated the switching response of Bcl-2 when we applied putative therapeutic interventions targeting these molecules (for example, an inhibitor of AC, antisense RNA targeting ICER and the overexpression of PDE3). The therapeutic effects of these targets were quite different from the effects obtained using β_1_-blockers, which expanded the survival range of the switching response profile to the right (Fig. 7c,g). Perturbations of ICER or PDE3 remarkably increased Bcl-2 expression only at high concentrations of ISO (>0.1 μM; Supplementary Fig. 11c,e), whereas the perturbation of AC significantly increased Bcl-2 expression at the whole concentration range of ISO (Supplementary Fig. 11d). Therefore, these results suggest that AC, ICER and PDE3 could be promising therapeutic targets for heart failure.

In this study, we present evidence that the strength of external stimuli for β-ARs plays an important role in the cellular decision for survival or death. On the other hand, Insel *et al*.^58^,^59^ provided evidence that the duration of the cAMP signal is important in the decision of cell survival or death by using S49 lymphoma. In their study, the incubation of S49 lymphoma cells with 8-CPT-cAMP (a membrane-permeable cAMP analogue) for 6 h showed significant resistance to apoptosis, but the apoptosis increased at 24-h time point. For further analysis, we conducted an additional simulation study using the protocol presented in the literature^58^. As a result, we found that Bcl-2 level was significantly increased during the initial period of treatment with cAMP analogue, but then it decreased with a longer treatment (Supplementary Fig. 13), suggesting that the duration of the cAMP signal is also important for cell fate determination. Taken together, the duration of cAMP signal as well as the strength of its stimulation might play an important role in the decision of cell survival or death.

**Figure 8:**
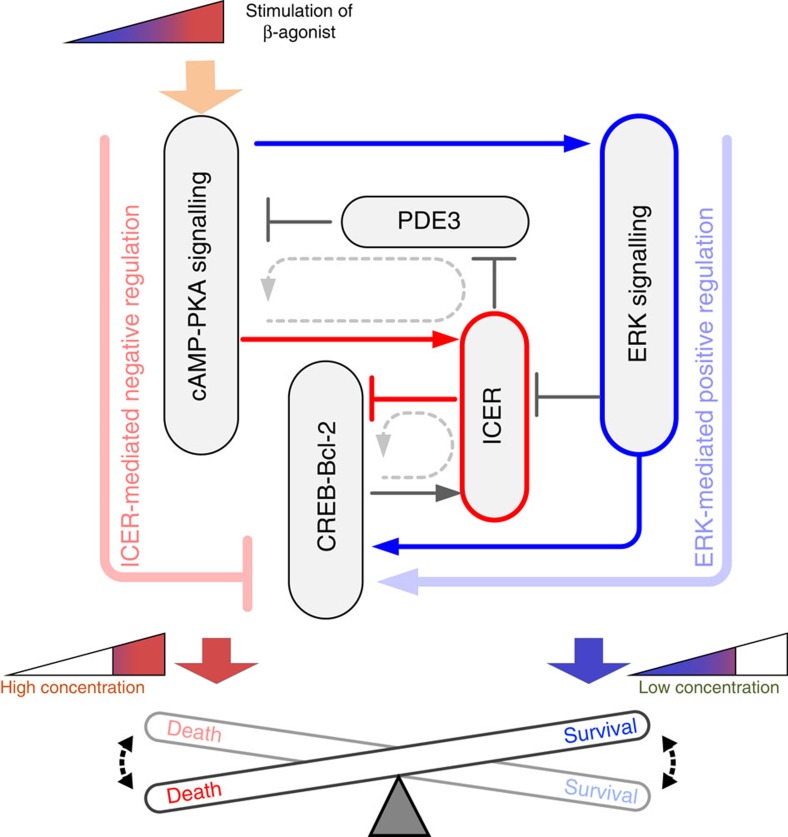
The core regulatory circuit is pivotal for the concentration-dependent cell fate determination. The eight essential regulatory links of the β-AR signalling network are primarily responsible for the switching response of Bcl-2 (thin solid lines). Among these, the ERK1/2 and ICER-mediated incoherent feed-forward loop is the core regulatory circuit that robustly generates the Bcl-2 switching response of cardiomyocytes, in which the ERK1/2 pathway at a low concentration range of ISO positively regulates Bcl-2 induction and thus cell survival ensues, whereas the PKA-ICER-CREB pathway at a high concentration range negatively regulates Bcl-2 induction and therefore promotes cell death.

Our results collectively suggest that the β-AR signalling network induces a switching response of Bcl-2 expression regulated by the ERK1/2 and ICER-mediated feed-forward loop to wide concentration ranges of β-AR agonists, and that this switching response determines the cell fate of cardiomyocytes (Fig. 8). This study provides a new insight into the concentration-dependent cell fate determination mechanism mediated by β-AR signalling in cardiomyocytes and suggests a novel therapeutic strategy for heart disease.

## Methods

### Mathematical modelling and parameter estimation

The model was developed using ODEs. The resulting ODE system consists of 32 state variables (see Supplementary Tables 1–4 for further details). We solved these ODEs by employing the Matlab built-in function, ode15s (ref. 60). To estimate the kinetic parameters of the ODE model, we minimized the sum of the squared difference between the experimental data and the simulated values using the genetic algorithm^61^ in Matlab Optimization Toolbox^62^ implemented on Window cluster composed of 260 CPUs in parallel (Supplementary Table 4). Details of the mathematical modelling, including model development, assumptions, estimated parameter sets and the system’s ODEs, can be found in Supplementary Tables 1–4 and Supplementary Note 1.

The simulation results of a mathematical model might highly depend on the parameter choice due to limited experimental data, resulting in an ill-posed parameter optimization problem. Thus, to evaluate the robustness of our results, we further carried out extensive simulations repetitively (*n*=30) over up to 30% random variation of parameter values selected from a uniform distribution and confirmed that the responses of the β-AR signalling network (that is, p-ERK1/2, p-CREB, expression of ICER, the activity of Ras, the formation of Grb2 and Shc complex, induction of cAMP and the activity of PKA) are robust to such parameter variations (Fig. 2).

The simplified ODE model composed of five nodes and eight essential regulatory links (Fig. 5b) was developed based on Michaelis–Menten-type functions (Supplementary Note 2). Non-dimensionalization of the model equations was carried out to reduce the number of parameters^39^ (Supplementary Note 4). A total of 10,000 sets of random parameters were generated to test the robustness of the model and parameter values were randomly sampled from a log uniform distribution in the ranges of *τ*=10–1000, *n*=1–10, *K*=0.01–1, *K*_*i*_=0.01–1 and β=0.1–10. These parameter ranges were chosen on the basis of the previous study^40^. All the simulations were carried out with zero initial conditions.

### Parameter sensitivity analysis

We carried out sensitivity analyses (LSA and GSA) of the network model to examine the influence of parameter perturbation on the Bcl-2 expression level quantitatively. For this purpose, we quantified the Bcl-2 expression by an integral of the Bcl-2 concentration over the observation time between 24 and 48 h for 1 μM ISO stimulation. The sensitivity score of LSA was obtained by the percent change in the quantity of interest caused by a 1% change of the model parameter^63^. In other words, the sensitivity score of LSA is defined by


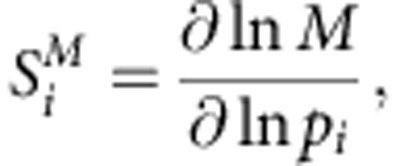


where *M* is the time-integrated Bcl-2 expression and *p*_*i*_ is the *i*th model parameter. The sensitivity score of GSA was obtained by using the partial rank correlation coefficient analysis that measures the correlation between the output (*M*) and model parameter (*P*_*j*_) while removing the correlation of the parameter of interest with other parameters^64^,^65^. To obtain the partial rank correlation coefficient between *P*_*j*_ and *M*, we first calculate the correlation coefficient *r*_*pj,m*_ between the two residuals 
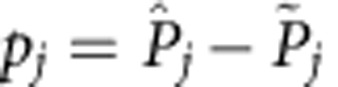
 and 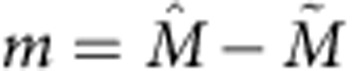, where 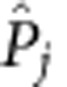 and 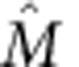 are the rank transformed *P*_*j*_ and *M*, respectively, obtained from the linear regression model defined as follows^64^:


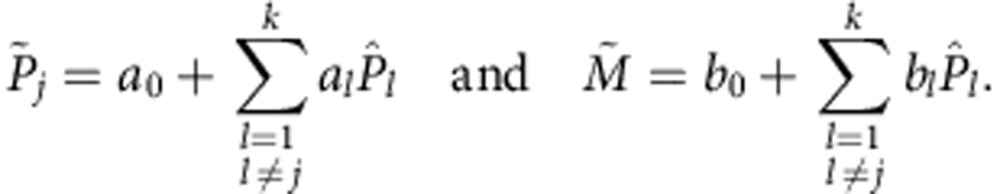


Thus


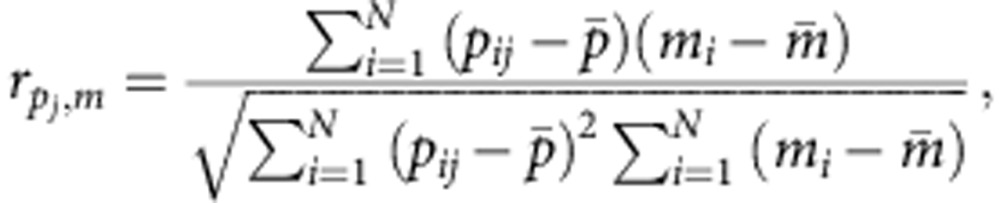


where *N* is the number of Sobol’s points sampled from the model parameter space, and 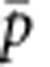 and 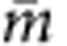 are the respective sample means. Note that we generated Sobol’s points by varying all parameter values within a 10-fold range around each nominal value. The negative sensitivity score indicates that the quantity of interest decreases if the parameter value increases, whereas the positive sensitivity score means that the quantity increases when the parameter value increases. A large score, whether positive or negative, implies a potentially significant effect on the Bcl-2 expression.

### Isolation and culture of adult rat ventricular myocytes

Adult rat ventricular myocytes were isolated from adult (10- to 14-week-old) male Sprague–Dawley rats as described with minor modifications^66^. In brief, hearts were excised from anesthetized (isoflurane inhalation) adult rats, mounted on a Langendorf apparatus and perfused retrogradely through the aorta with oxygenated Ringer’s solution of the following composition: 125 mM NaCl, 5 mM KCl, 25 mM HEPES, 2 mM KH_2_PO_4_, 1.2 mM MgSO_4_, 5 mM pyruvate, 11 mM glucose, 5 mM creatin, 5 mM L-carnitine and 5 mM taurine (pH 7.4 adjusted with NaOH). Initial perfusion was for 5 min with Ringer’s solution containing 1 mM CaCl_2_ followed by another 15 min perfusion with calcium-free Ringer’s solution. Calcium-free Ringer’s solution containing 230 U ml^−1^ Collagenase Type 2 (Worthington) and 60 nM (0.4 mg ml^−1^) hyaluronidase (Sigma) was recirculated through the heart for 30 min. Final perfusion was for 1 min with Ringer’s solution containing 4% BSA (Bovogen) and 10 mM 2,3-butanedione monoxime (Sigma). The cannulus was removed from the heart and the ventricles were cut away and diced.

Myocytes were filtered through a 100 μm Cell Strainer (BD Biosciences), and then CaCl_2_ was added to the myocytes to the final concentration of 1.8 mM for 10 min. Myocytes were plated on 13 pM (11 μg ml^−1^) laminin (BD Biosciences)-coated tissue culture dish (Corning) at the density of 10^4^ cells cm^−2^ and incubated at 37 °C and 5% CO_2_ in M199 medium (Sigma) containing 25 mM HEPES, 2.2 g l^−1^ antibiotics(Gibco), 2.5 mM taurine, 2.5 mM carnitine and 2.5 mM creatine. After 2-h incubation, unattached cells were removed by washing with same media.

All animal experiments were carried out in observance with Gwangju Institute of Science and Technology Animal Care and Use Committee guidelines.

### Cell death assay by live-cell imaging

Myocytes were seeded in a 12-well tissue culture plate and allowed to adhere for 2 h. ISO was added to myocytes at the indicated concentrations for 12 h and apoptosis was induced by adding 3.5 μM ionomycin (Sigma) or 100 μM H_2_O_2_. Live-cell images of myocytes were captured at 10-min intervals for 5 h at 37 °C in M199 medium by using automated microscopy (IN Cell Analyzer 1000 HCS system, GE). Cell survival rate was assessed by counting the number of rod-shaped cells containing intact sarcomeric structure before and after the apoptosis induction. The experiment was conducted using more than three independent primary cultures.

### Analysis of cell death by ELISA

Cytoplasmic accumulation of histone-associated DNA fragments (mono- and oligonucleosomes) was quantified by the Cell Death Detection ELISA PLUS kit (Roche Diagnostics) by following the manufacturer’s instructions.

### Western blot analysis

After treatment with drugs, cells were lysed in 1% SDS lysis buffer containing protease inhibitor cocktail, Na_3_VO_4_ and NaF. Proteins were estimated by using BCA assay kit (Thermo scientific) and 40 μg of proteins were resolved electrophoretically on 10–15% SDS polyacrylamide gels, transferred to polyvinylidene fluoride membrane. The blots were probed with the following antibodies (see the list below) and detected by chemiluminescent substrate (Thermo scientific) and Biomolecular imager, ImageQuant LAS 4000 mini (GE). The band intensities were quantified using NIH ImageJ software.

The following primary antibodies were used for western blot analysis: phospho-CREB (S-133; dilution 1:1000) (#9197), CREB (#9198; dilution 1:1000), ERK1/2 (#9102; dilution 1:1000), phospho-ERK1/2 (#9101s; dilution 1:1000), Bax (#2772; dilution 1:1000) were purchased from Cell Signaling Technology. Bcl-2 (sc-7382; dilution 1:500), ICER (sc-440; dilution 1:500), α-tubulin (sc-5286; dilution 1:1000) and catalytic subunit of PKA (sc-903; dilution 1:500) were purchased from Santa Cruz Biotechnology. GAPDH (glyceraldehyde-3-phosphate dehydrogenase) from Lab Frontier (dilution 1:5000) and rabbit anti-ICER antibody (dilution 1:10000) from Dr Carlos Molina (Montclair State University) were used for the study.

### RNA extraction and quantitative real-time PCR (qRT–PCR)

Total RNA extraction, first-strand cDNA synthesis and qRT–PCR were performed as we described previously^23^. In brief, approximately, 10^5^ cells were used for the extraction of total RNA with 1 ml Trizol Reagent (Invitrogen, Carlsbad, CA) following the manufacturer’s instructions. First-strand cDNA was synthesized from 500 ng of total RNA with random hexamer and oligo dT using PrimeScript RT reagent Kit (Takara, Japan) following the manufacturer’s instructions. qRT–PCR assays using StepOnePlus Real-Time PCR system (Applied Biosystems) were performed using SYBR Premix Ex TaqTM (TaKaRa) under the following two-step conditions: denaturation at 95 °C for 3 s; and annealing and extension at 60 °C for 40 s, for a total of 40 cycles. GAPDH was used as an endogenous reference to assess the relative level of mRNA transcript.

The following primers were used for PCR analysis:

GAPDH, 5′- ATGTTCCAGTATGACTCCACTCACG -3′ (sense)

and 5′- GAAGACACCAGTAGACTCCACGACA -3′ (antisense)

Bcl-2, 5′- TGAACCGGCATCTGCACAC -3′ (sense)

and 5′- CGTCTTCAGAGACAGCCAGGAG -3′ (antisense)

Xiap, 5′- CAAGTGAAGACCCTTGGGAACA -3′ (sense)

and 5′- TTCTTGCACCATAGGATTCTGGA -3′ (antisense)

cIAP1, 5′- GTTGGGAACCCGGAGATGAC -3′ (sense)

and 5′- CCTTCATCCGTATCAAGAACTCACA -3′ (antisense)

cIAP2, 5′- CAAGTTCAAGCTGGTTACCCTCATC -3′ (sense)

and 5′- AGGTGTGTTCATCATCACTGCATC -3′ (antisense)

Bim, 5′- TTGCCAGGCCTTCAACCATTA -3′ (sense)

and 5′- CAGCTCCTGTGCGATCCGTA -3′ (antisense)

PDE3a, 5′- TTCATGCTTTGGAGATCGGCTAC -3′ (sense)

and 5′- AGGAATCGGCTGTGTTGTGAGATAC -3′ (antisense)

### siRNA transfection

Control siRNA (siControl) and ICER siRNA (siICER) oligonucleotides were synthesized by BIONEER Corporation in a sense-antisense duplex form. For siRNA transfection, 1 μl of a 100-μM stock of siRNAs and 4 μl of DharmaFECT (Thermo Scientific) were each incubated separately with 50 μl Opti-MEM (Invitrogen) for 5 min, mixed together for 20 min at room temperature, and then 100 μl was applied to the adult cardiomyocyte on the 35-mm culture dish (final siRNA concentration was 100 nM). Three hours after transfection, cardiomyocytes were stimulated with various concentrations of ISO.

Previously reported siRNA sequences were used in this study^41^:

siControl, 5′- GAGUACUUAAGGAUGACUAUU(dTdT) -3′ (sense)

and 5′- AAUAGUCAUCCUUAAGUACUC(dTdT) -3′ (antisense)

siICER, 5′- CUUAUAGAGGAGCUUGAAA(dTdT) -3′ (sense)

and 5′- UUUCAAGCUCCUCUAUAAG(dTdT) -3′ (antisense)

### Reagents

RP-cAMPS was purchased from BioLog, and ICI 118,551 was purchased from Santa Cruz Biotechnology. All other reagents including ISO, PD98059, (±)-Metoprolol and cycloheximide were from Sigma.

### Statistical analysis

Results from at least three independent experiments are expressed as mean±s.e.m. (or mean±s.d.). Comparisons between groups were performed by Student’s two-tailed *t*-test for experiments. Probability values <0.05 were considered statistically significant. All analyses were performed with SigmaPlot 12 software (Systat Software Inc.).

## Author contributions

S.-Y.S., T.K., K.-H.C. and D.H.K. designed the study. S.-Y.S., T.K., H.-S.L., J.H.K. and K.-H.C. developed the mathematical models. S.-Y.S., H.-S.L., J.H.K. and K.-H.C. conducted simulations and analysed the simulation results. T.K. performed most of the experiments and analysed the experimental data. J.Y.L. assisted the experiments. S.-Y.S., T.K., H.-S.L., J.H.K., K.-H.C. and D.H.K. wrote the manuscript. K.-H.C. and D.H.K supervised the study.

## Additional information

**How to cite this article:** Shin, S.-Y. *et al*. The switching role of β-adrenergic receptor signalling in cell survival or death decision of cardiomyocytes. *Nat. Commun.* 5:5777 doi: 10.1038/ncomms6777 (2014).

## Supplementary Material

Supplementary Information Supplementary Figures 1-14, Supplementary Tables 1-6, Supplementary Notes 1-4 and Supplementary References

Supplementary Movie 1 Merged movie simultaneously showing cardiomyocytes under the four different experimental conditions (Control, 500 ȝM RP-cAMPS + 1 ȝM ISO, 10 ȝM RP-cAMPS + 1 ȝM ISO, 1 ȝM ISO). Cardiomyocytes pre-incubated with or without RP-cAMPS (at 10 or 500 ȝM for 2 hours) and treated with 1 ȝM ISO were imaged for 12 hours with 1-hour interval using the automated microscope. The analyzed results for the survival rate of the experimental groups are shown in Supplementary Fig. 3.

Supplementary Movie 2 Representative time-lapse images of control cardiomyocytes. Cardiomyocytes were imaged for 5 hours with 5 minutes intervals using an automated microscope (IN Cell Analyzer 1000 HCS system, GE). Most cardiomyocytes maintained their healthy morphological features (rod-shaped cell body and intact sarcomeric structure) although few of them were disrupted (Fig. 4g).

Supplementary Movie 3 Representative time-lapse images of H2O2-treated cardiomyocytes. Cardiomyocytes treated with H2O2 were imaged for 5 hours with 5 minutes intervals using the automated microscope. Most of cardiomyocytes were eventually disrupted by the treatment of H2O2 (Fig. 4g).

Supplementary Movie 4 Representative time-lapse images of 10 ȝM ISO plus H2O2-treated cardiomyocytes. Cardiomyocytes pre-incubated with 10 ȝM ISO for 12 hours followed by treatment with H2O2 were imaged for 5 hours with 5 minutes intervals using the automated microscope. The disruption of the cardiomyocytes was slower and the number of the rod-shaped cardiomyocyte (with intact sarcomere) was larger than the H2O2-only treated group (Fig. 4g)

Supplementary Movie 5 Representative time-lapse images of 10 ȝM ISO plus H2O2-treated cardiomyocytes. Cardiomyocytes pre-incubated with 10 ȝM ISO for 12 hours followed by treatment with H2O2 were imaged for 5 hours with 5 minutes intervals using the automated microscope. Most cardiomyocytes were eventually disrupted, but the speed of disruption was similar to the H2O2-only treated group (Fig. 4g).

Supplementary Movie 6 Representative time-lapse images of ionomycin-treated cardiomyocytes. Cardiomyocytes treated with ionomycin were imaged for 5 hours with 5 minutes intervals using the automated microscope. Sarcomeric structures of most cardiomyocytes were disrupted by the treatment of ionomycin (Fig. 4h).

Supplementary Movie 7 Representative time-lapse images of 10 ȝM ISO plus ionomycin-treated cardiomyocytes. Cardiomyocytes pre-incubated with 10 ȝM ISO for 12 hours followed by treatment with ionomycin were imaged for 5 hours with 5 minutes intervals using the automated microscopy. Disruption of cardiomyocytes was slower and the number of rod-shaped cardiomyocytes (with intact sarcomere) was larger than the ionomycin-only treated group (Fig. 4h).

Supplementary Movie 8 Representative time-lapse images of 10 ȝM ISO plus ionomycin-treated cardiomyocytes. Cardiomyocytes pre-incubated with 10 ȝM ISO for 12 hours followed by treatment with ionomycin were imaged for 5 hours with 5 minutes intervals using the automated microscopy. Disruption of cardiomyocytes was slower and the number of rod-shaped cardiomyocytes (with intact sarcomere) was larger than the ionomycin-only treated group (Fig. 4h).

Supplementary Movie 9 Morphological changes due to H2O2-induced cell death. In H2O2-induced apoptotic cardiomyocytes, the sarcomeric structure was disrupted and eventually the cell body of cardiomyocytes became rounded.

Supplementary Movie 10 Morphological changes due to ionomycin-induced cell death. In ionomycininduced apoptotic cardiomyocytes, the sarcomeric structure was disrupted and eventually the cell body of cardiomyocytes became rounded.

Supplementary Movie 11 Merged movie simultaneously showing cardiomyocytes under the two different experimental conditions (siControl + 1 μM ISO, siICER + 1 μM ISO) Cardiomyocytes transfected with siControl or siICER and then treated with 1 μM ISO were imaged for 12 hours with 1-hour interval using the automated microscope. siICER group shows higher survival rate than siControl group. The analyzed results of the survival rate of those groups are shown in Fig. 6e.

Supplementary Movie 12 Merged movie simultaneously showing cardiomyocytes under the two different experimental conditions (1 nM ISO, PD98059 + 1 nM ISO). Cardiomyocytes pre-incubated with or without PD98059 and then treated with 1 nM ISO were imaged for 12 hours with 1-hour interval using the automated microscope. Control group shows higher survival rate than PD98059-treated group. The analyzed results of the survival rate of those groups are shown in Fig. 6h

Supplementary Movie 13 Merged movie simultaneously showing cardiomyocytes under the two different experimental conditions (10 nM ISO, PD98059 + 10 nM ISO) Cardiomyocytes pre-incubated with or without PD98059 and then treated with 10 nM ISO were imaged for 12 hours with 1-hour interval using the automated microscope. Control group shows higher survival rate than PD98059-treated group. The analyzed results of the survival rate of those groups are shown in Fig. 6h.

Supplementary Movie 14 Merged movie simultaneously showing cardiomyocytes under the two different experimental conditions (100 nM ISO, metoprolol + 100 nM ISO) Cardiomyocytes pre-incubated with or without metoprolol and then treated with 100 nM ISO were imaged for 12 hours with 1-hour interval using the automated microscope. Metoprolol-treated group shows higher survival rate than the control group. The analyzed results of the survival rate of those groups are shown in Fig. 7i.

Supplementary Movie 15 Merged movie simultaneously showing cardiomyocytes under the two differentexperimental conditions (1 μM ISO, metoprolol + 1 μM ISO). Cardiomyocytes pre-incubated with or without metoprolol and then treated with 1 μM ISO were imaged for 12 hours with 1-hour interval using the automated microscope. Metoprolol-treated group shows higher survival rate than the control group. The analyzed results of the survival rate of those groups are shown in Fig. 7i. http://sbie.kaist.ac.kr/KHC/Video_15_Control_vs_Meto_(ISO_1uM).avi
