# Supplementary material for: The switching role of β-adrenergic receptor signalling in cell survival or death decision of cardiomyocytes
Source: Nat Commun. 2014 Dec 17;5:5777. doi: 10.1038/ncomms6777 (PMC4284638; doi:10.1038/ncomms6777)
Supplement: Supplementary Information — Supplementary Figures 1-14, Supplementary Tables 1-6, Supplementary Notes 1-4 and Supplementary References [file ncomms6777-s1.pdf]

I. Supplementary Figures

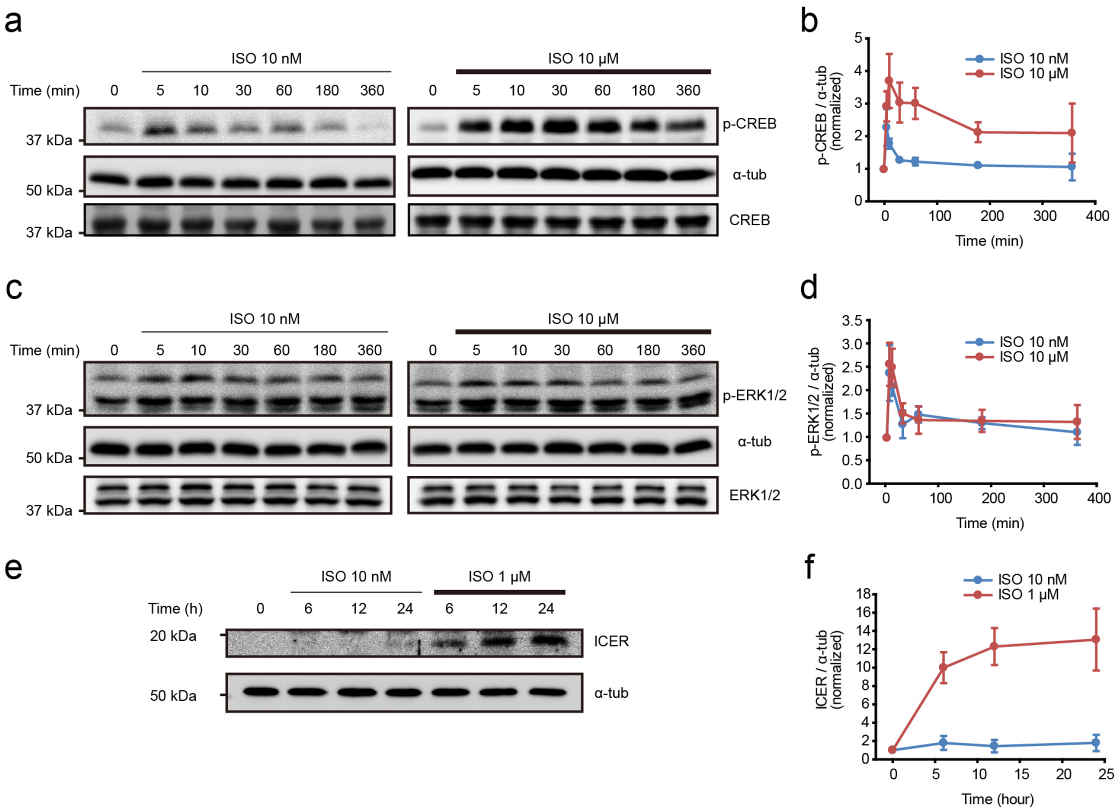

Supplementary Fig. 1. Response time courses of the major signalling molecules to the low or high concentrations of ISO. (**a**,**c** and **e**) Representative immunoblots for CREB, p-CREB, ERK1/2, p-ERK1/2, ICER and α-tubulin (loading control). Note that total ERK1/2 and CREB were not changed by the low or high concentration of ISO. (**b**, **d** and **f**) Quantitative graphs for the ratio of p-CREB, p-ERK1/2 and ICER to the loading control in the presence of lower (10 nM) or higher (1-10 μM) ISO compared to the non-stimulation control. The data represent mean ± SEM, n≥3 biological and technical replicates (independent culture preparations).

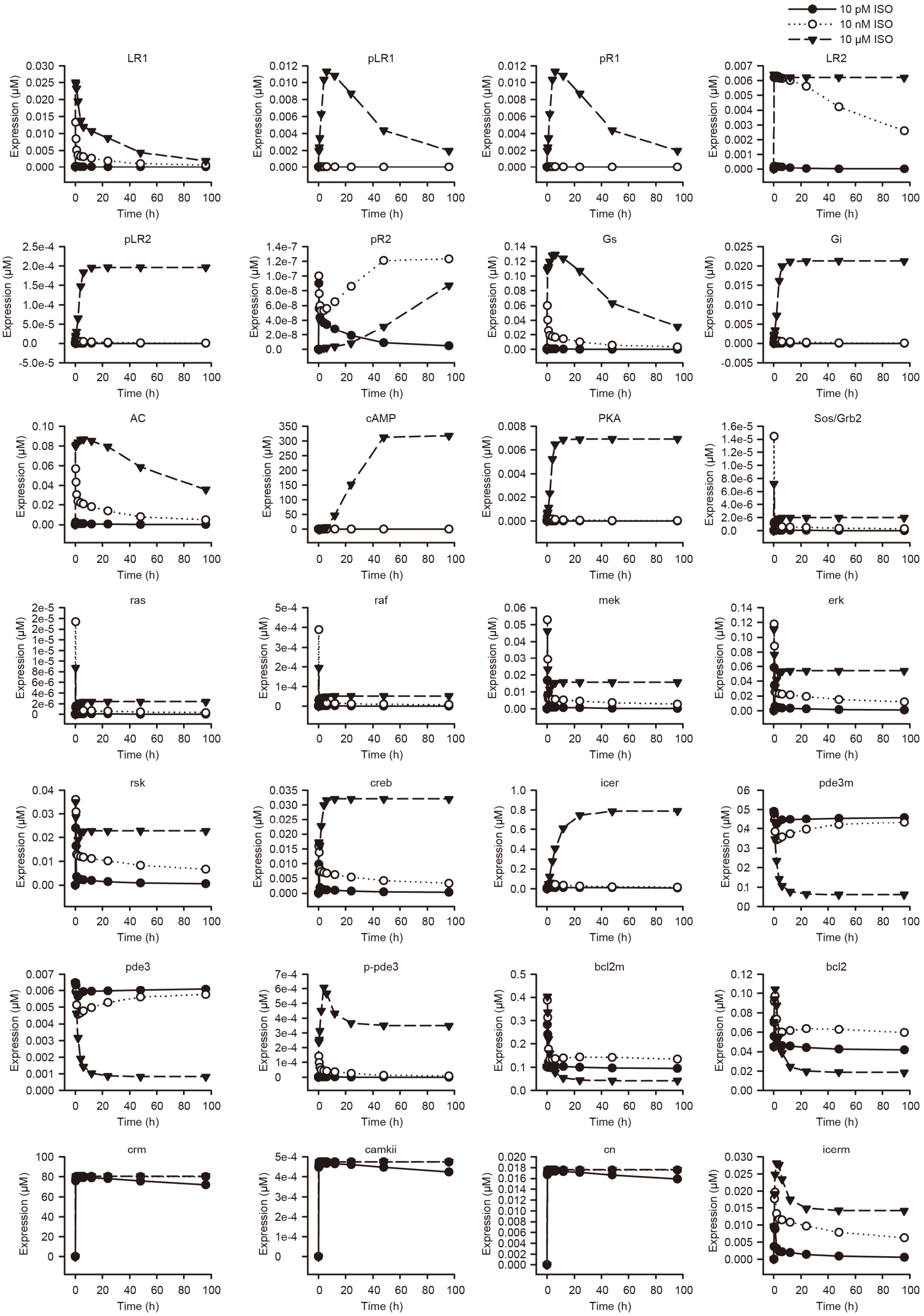

Supplementary Fig. 2. The temporal simulation profiles of all the signalling components in the model. Graphs show the simulation results of the expression patterns of all signalling components included in the model (at three ISO concentrations: 10 pM, 10 nM and 10 μM for 96 hours). LR1, ligand-bound β_1-AR; pLR1, phosphorylated ligand-bound β_1-AR; pR1, phosphorylated β_1-AR; LR2, ligand-bound β_2-AR; pLR2, phosphorylated ligand-bound β_2-AR; pR2, phosphorylated β_2-AR; crm, calcium regulatory machinery.

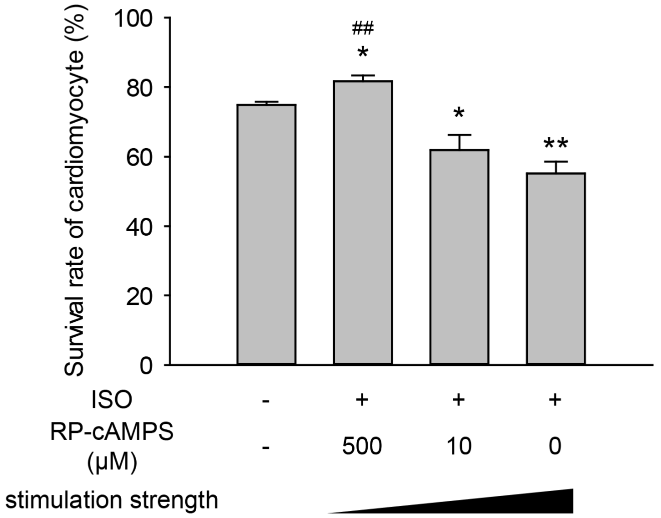

Supplementary Fig. 3. The survival rate of cardiomyocytes depending on the stimulation strength of β-AR. Cardiomyocytes were pre-incubated with the indicated concentrations of a PKA inhibitor (RP-cAMPS) for 2 hours prior to incubation with 1 μM ISO for 12 hours. The survival rate was assessed by live-cell imaging and counting the healthy cells before and after ISO stimulation. The data represent means ± SEM, n = 3 biological and technical replicates (independent culture preparations). *, $p < 0.05$; **, $p < 0.01$ versus control group; ##, $p < 0.01$ versus ISO-treated group; Student's t-test.

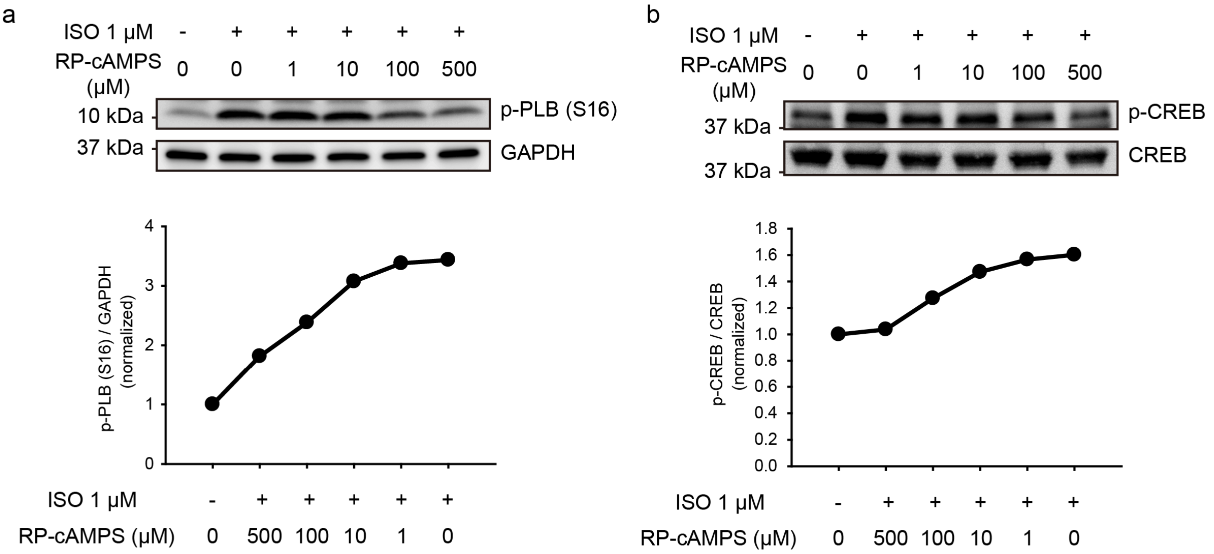

Supplementary Fig. 4. Phosphorylation of phospholamban (PLB) or CREB increased along with the increasing cAMP level. (**a** and **b**) Cardiomyocytes were pre-incubated with the indicated concentrations of PKA inhibitor (RP-cAMPS) for 2 hours before 12 hours of incubation with 1 μM ISO to measure p-phospholamban (PLB) on S16 (a) and p-CREB (b). Quantitation of the phosphorylation levels of PLB and CREB with the corresponding loading controls (lower panel). Note that the concentrations of RP-cAMP were rearranged in the order of increased signalling flux through the cAMP-PKA signalling module.

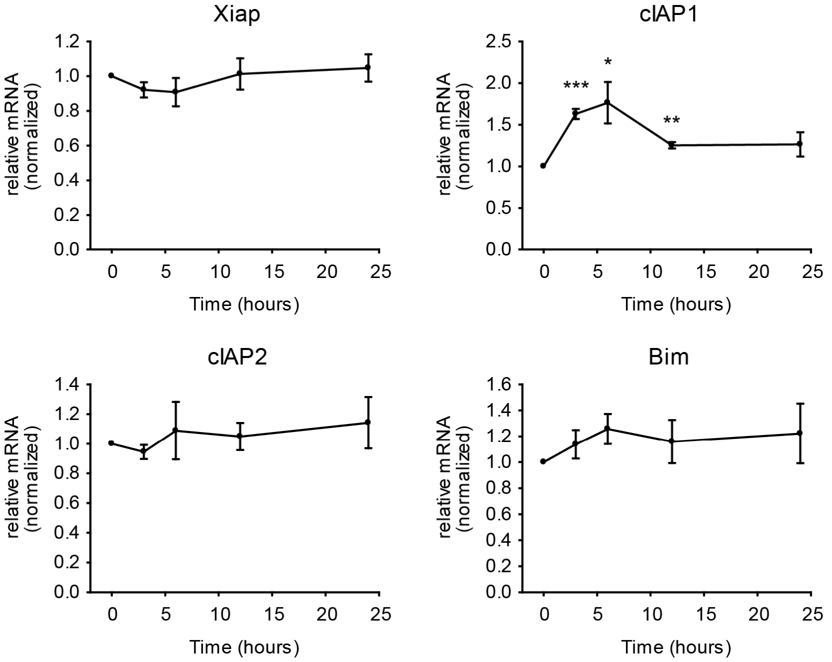

Supplementary Fig. 5. The temporal profiles of cAMP-related apoptotic molecules in response to ISO stimulation. Cardiomyocytes were stimulated with 1 μM ISO, and the mRNA level of cAMP-related apoptotic molecules were measured over time by qRT-PCR. The expression levels of Xiap, cIAP2, and Bim did not show any significant change in response to ISO. cIAP1 was transiently increased for the initial 12 hours and then decreased to its basal level. The data represent means ± SEM, n≥3 biological and technical replicates (independent culture preparations). *, $p < 0.05$; **, $p < 0.01$; ***, $p < 0.001$ versus control group; Student's t-test.

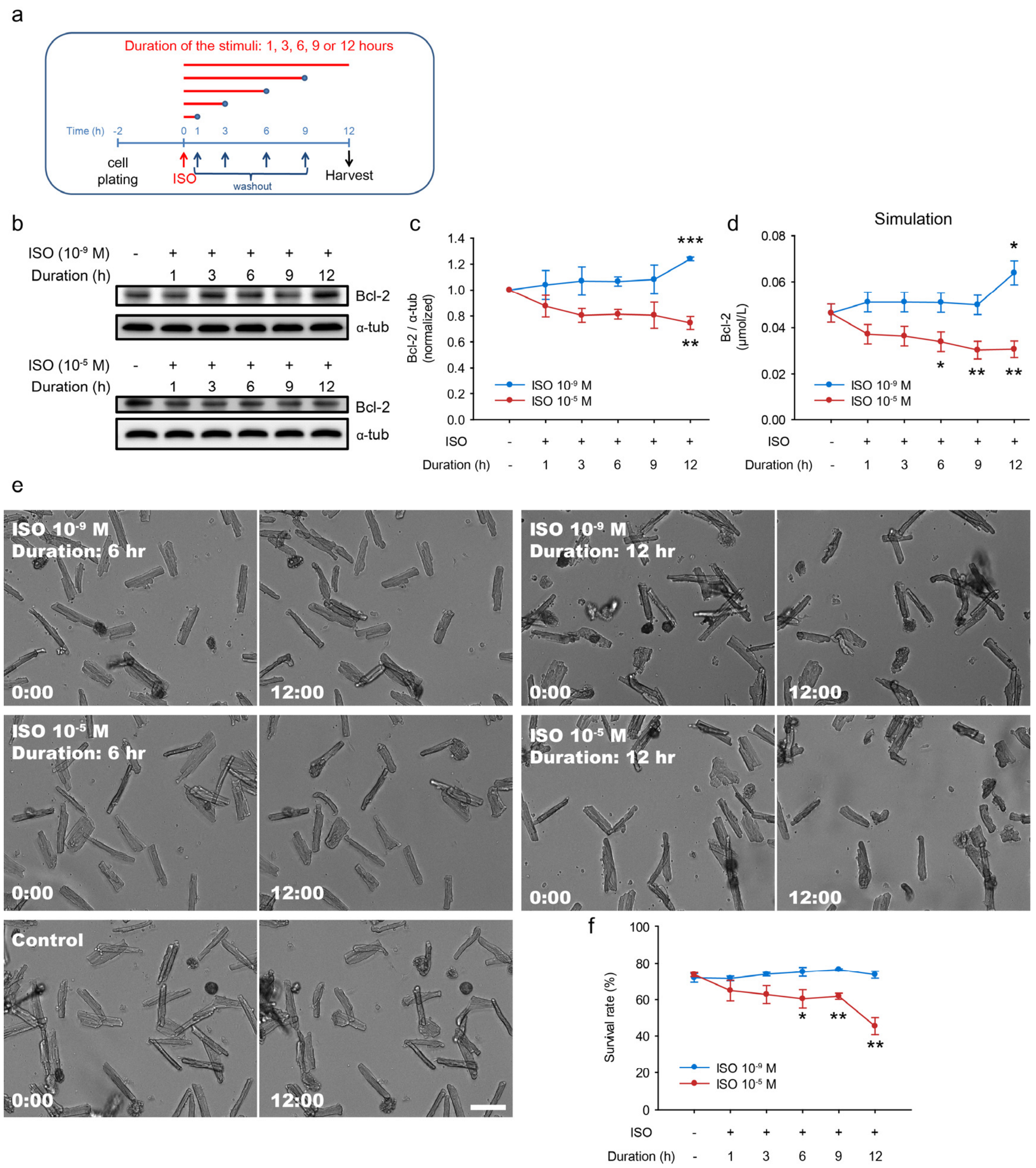

Supplementary Fig. 6. The effect of the duration of β-AR stimuli on the cellular decision for survival or death. To examine the effect of the duration of the stimuli on the Bcl-2 expression level and cell viability, experiments and simulation were performed. (**a**) A schematic diagram showing the protocol of investigating the effect of the duration of β-AR stimuli on the cellular decision for survival or death. Isolated adult cardiomyocytes were incubated with indicated concentrations of ISO for 1, 3, 6, 9 or 12 hours. At the end of stimulation, ISO was washed out. Cardiomyocytes were lysed for western blotting analysis or imaged for calculating the survival rate at 12 hours from the start of ISO treatment. (**b-c**) Representative Bcl-2 and α-tubulin (loading control) immunoblots and the semi-quantified data showed that the effect of ISO on the cellular decision for survival or death at the low or high concentration was enhanced along with the washout time, respectively (the longer the stimulation, the stronger the effect). The data represent means ± SEM, n ≥ 4 biological and technical replicates (independent culture preparations). (**d**) Both simulation (d) and experimental (c) results showed the linear correlation between the duration of stimulation and the enhancement of the effect on the expression level of Bcl-2. On the other hand, the cell fate determination does not change by the duration of the stimuli. (**e-f**) The survival rate was assessed by live-cell imaging. Data represent means ± SEM, n = 3 biological and technical replicates (independent culture preparations), n=20 for simulation (d). *, $p < 0.05$; **, $p<0.01$; ***, $p<0.001$ compared to control group; Student's t-test.

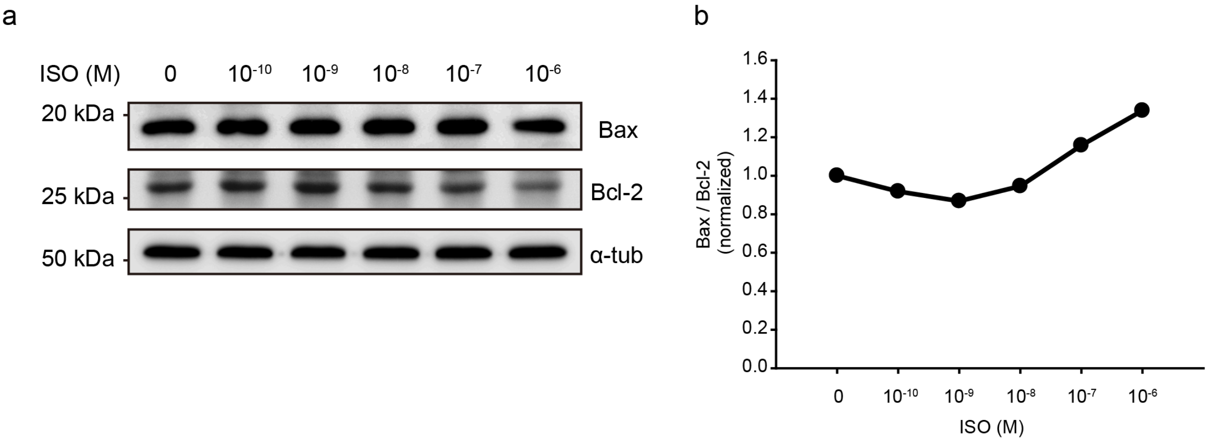

Supplementary Fig. 7. ISO-dependent expression levels of Bax and Bcl-2.
(**a**) Representative immunoblots for Bax, Bcl-2, and α-tubulin (loading control) expressed with 12 hours of incubation at the indicated concentrations of ISO. The Bcl-2 expression level obviously increased at the concentration range of 10^{-10}-10^{-9} M, but further decreased to below its basal level at the concentration range of 10^{-7}-10^{-6} M, but the Bax expression level was not changed significantly. (**b**) The ratio of Bax to Bcl-2 quantified from the immunoblots shown in (a).

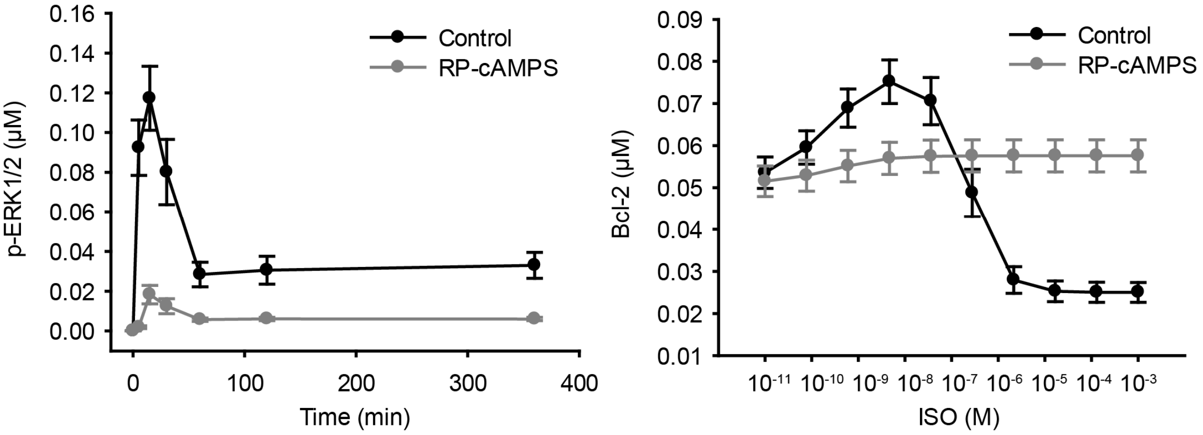

Supplementary Fig. 8. PKA plays an important role in increasing the level of Bcl-2 expression at the low concentration range of ISO through ERK1/2 activities. Simulation results show that the PKA inhibitor (RP-cAMPS) significantly suppressed ERK1/2 activity (left panel) and the switching profile of Bcl-2 disappears after the PKA inhibition (right panel). The data represent mean ± SEM for the repetitive simulations (n = 20) over up to 20% random variation of parameter values.

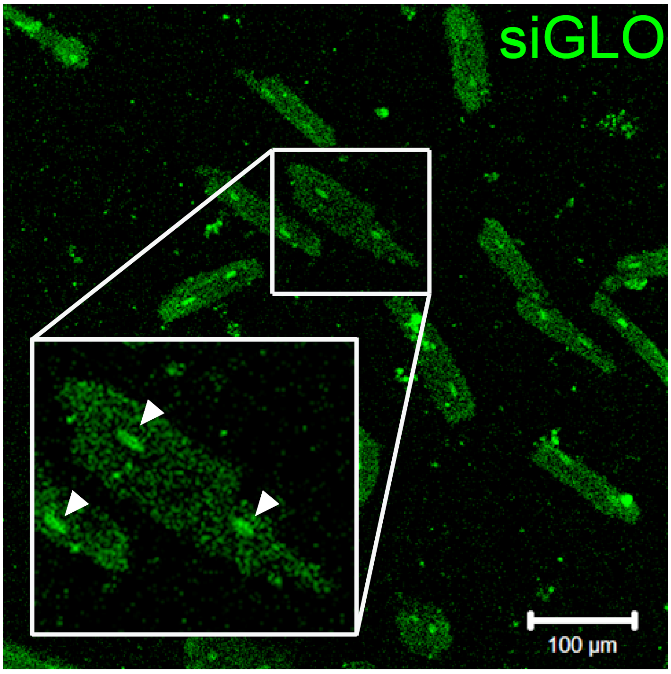

Supplementary Fig. 9. The efficiency of siRNA transfection in adult cardiomyocytes. To evaluate the efficiency of siRNA transfection into adult cardiomyocytes, siGLO Transfection Indicators (Thermo Scientific Inc.) was used. 24 hours after transfection, cardiomyocytes were imaged using a confocal microscope. Most of the rod-shape and multi-nuclei cardiomyocytes contain siGLO (green) signal, which is enriched in the nuclear region (arrow heads). Scale bar = 100 µm.

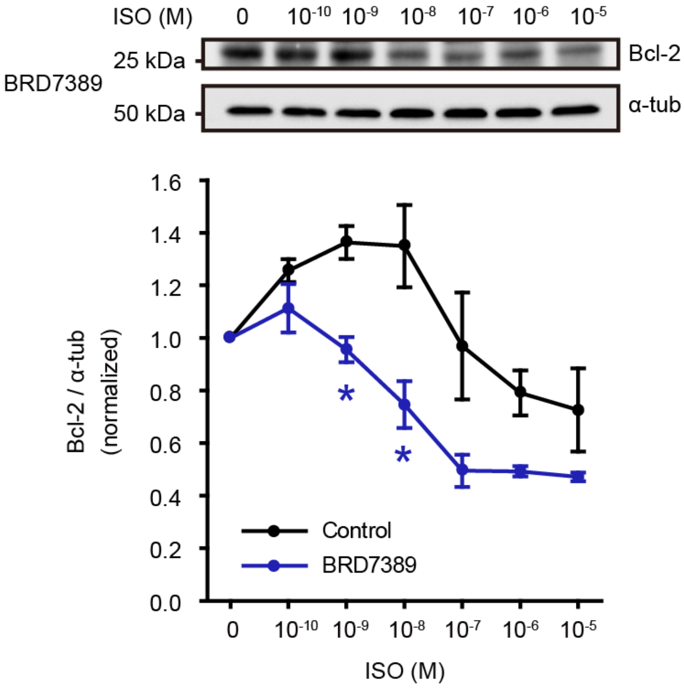

Supplementary Fig. 10. Validation of the role of ERK1/2-mediated feed-forward loop using a RSK inhibitor. Cardiomyocytes were stimulated with the indicated concentrations of ISO in the presence of BRD7389. Representative immunoblots and plots of semi-quantification showing Bcl-2 expression at the indicated ISO concentration determined in the presence or absence of BRD7389. Data represent means ± SEM, n = 4 biological and technical replicates (independent culture preparations). The control data were taken from Fig. 6i. *, $p < 0.05$; Student's t-test.

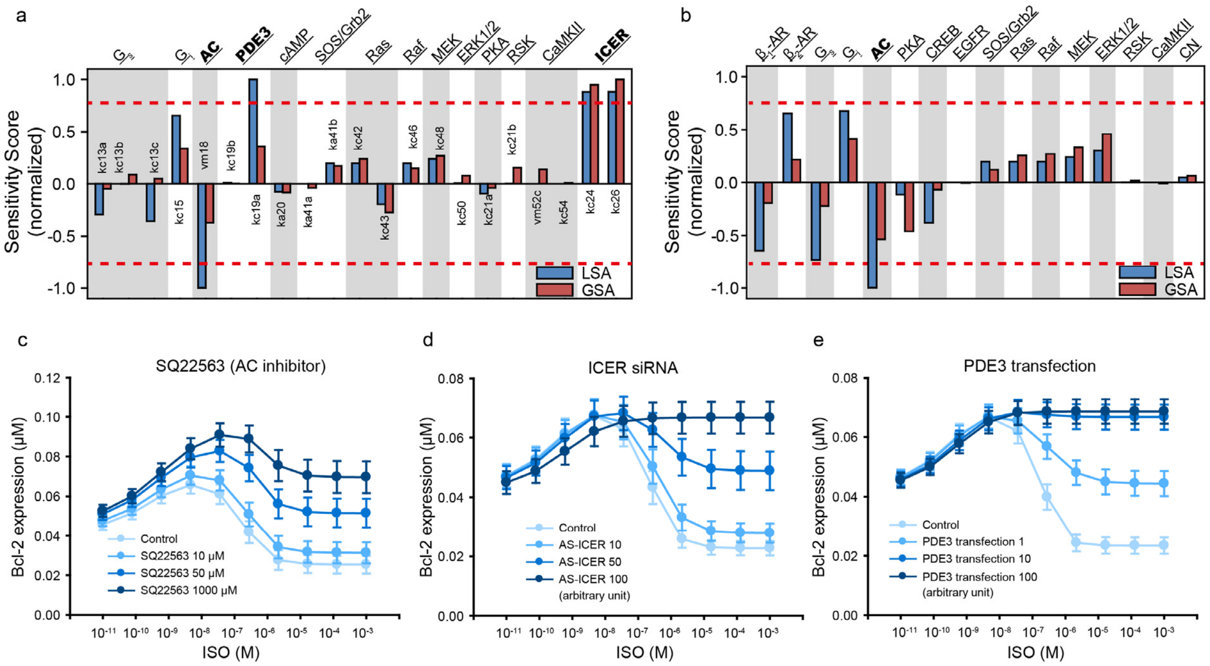

Supplementary Fig. 11. Potential therapeutic targets were predicted from the sensitivity analysis of the kinetic parameters and the total concentrations. (**A**) Before performing the sensitivity analysis, we had screened all the kinetic parameters and identified 21 parameters that could be the potential therapeutic targets by surveying the previous experimental data (see Supplementary Table 1 for details). The model parameters associated with AC, PDE3 and ICER have significant effects on the time-integrated Bcl-2 expression. The blue and red bars represent the results of LSA and GSA, respectively. (**B**) The sensitivity analysis of the total concentrations of signalling proteins. AC has a significant effect on the time-integrated Bcl-2 expression. Perturbation of AC **(C)** or PDE3 **(E)** remarkably increases the Bcl-2 expression only at high concentrations of ISO. (**D**) Perturbation of ICER significantly increases the Bcl-2 expression over all the concentration ranges. The data represent mean ± SEM for the repetitive simulations (n = 20) over up to 20% random variation of parameter values.

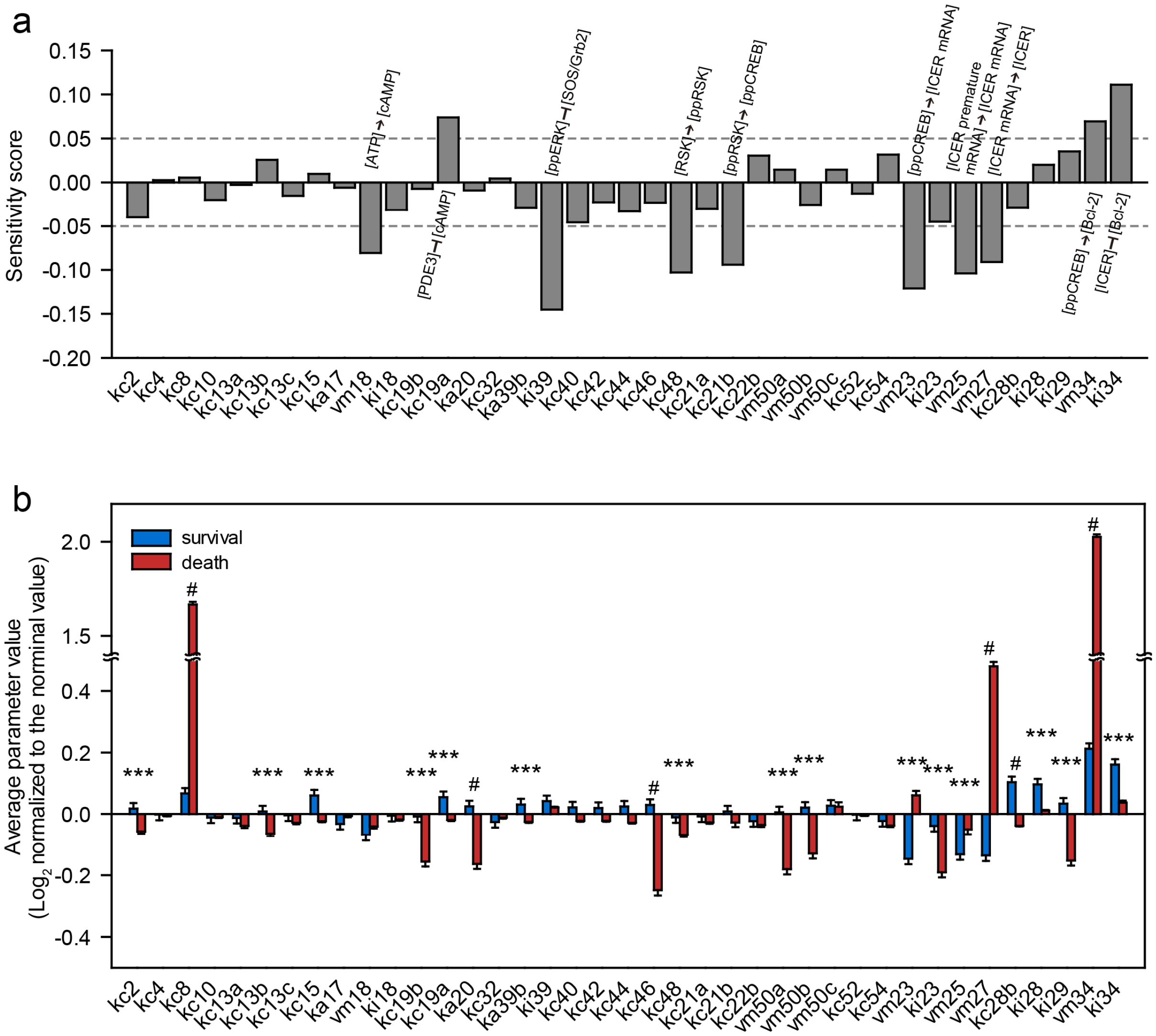

Supplementary Fig. 12. Sensitivity analysis of the network parameters reveals important parameters in controlling the Bcl-2 expression. (**a**) Parameter values were randomly sampled from a log uniform distribution in the range of 10-fold variation of the nominal values and the 6,346 (out of 10,000) sets of parameters that generate the Bcl-2 switching response were collected and analyzed. The sensitivity analysis was carried out based on the partial rank correlation coefficient (PRCC) method. (**b**) Parameter values were randomly sampled from a log uniform distribution in the range of 10-fold variation of the nominal values and parameter sets were clustered into two groups leading to either 'survival-only' or 'death-only' response which was defined on the basis of Bcl-2 response profile. The parameter values were represented by their normalized values with respect to their nominal values. Data represent means ± SEM, ***, $p<0.001$; #, $p<10^{-15}$; Parameter values of two groups were statistically compared with each other using Student's t-test.

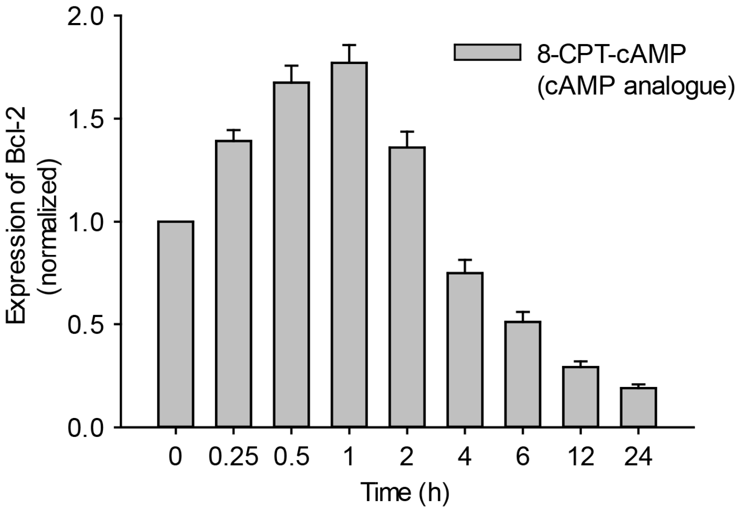

Supplementary Fig. 13. The switching effect of cAMP signal on cell fate-determination depending on the stimulation durations. The simulation results show that Bcl-2 level was increased during the initial period of treatment with a cAMP analogue (8-CPT-cAMP), but then it was decreased for a longer treatment. The data represent mean ± SEM for the repetitive simulations (n = 20) over up to 30% random variation of parameter values.

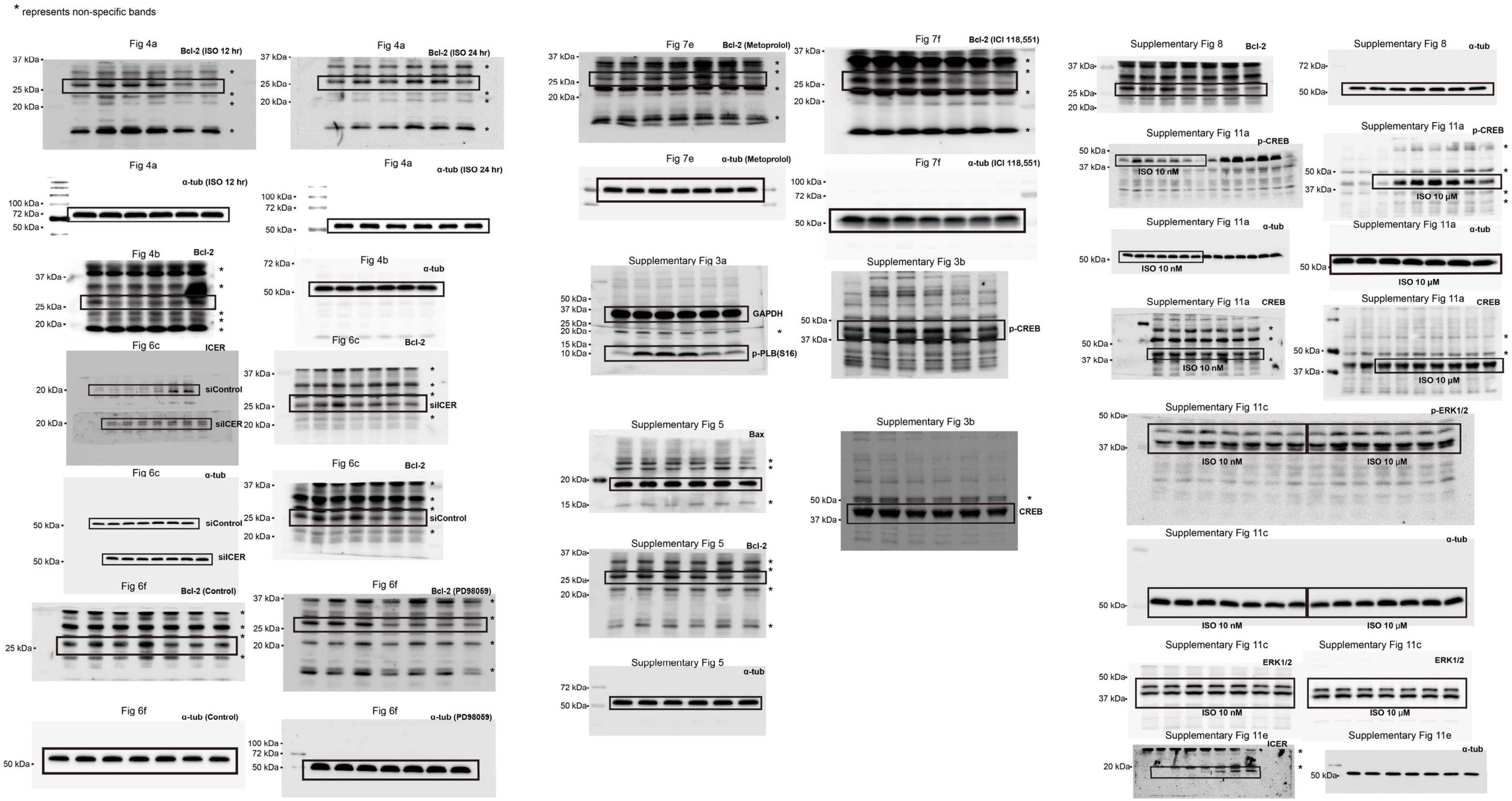

Supplementary Fig. 14. Uncropped western blots related to Figs. 4, 6, 7, and Supplementary Figs. 1, 4, 6, 7 and 10. * represents non-specific bands.

II. Supplementary Tables

Supplementary Table 1. Reactions/Processes

No	Reaction/Process	Equation
ISO-β_1AR interaction		
v1	[ISO] + [R1] ↔ [LR1]	ka1[ISO][R1][1)] – kd1[LR1]
v1a	[Meto] + [R1] ↔ [MetoR1]	ka1a[Meto][R1] – kd1a[MetoR1]
v2	[LR1] → [pLR1]	kc2[LR1]([PKAC]/(1 + [RpcAMP]/ki_rpcamp))
v3	[pLR1] → [LR1]	kc3[pLR1]
v4	[R1] → [pR1]	kc4[R1]([PKAC]/(1 + [RpcAMP]/ki_rpcamp))
v5	[pR1] → [R1]	kc5[pR1]
v6	[ISO] + [pR1] → [pLR1]	ka6[ISO][pR1] – kd6[pLR1]
ISO-β_2AR interaction		
v7	[ISO] + [R2] ↔ [LR2]	ka7[ISO][R2][2)] – kd7[LR2]
v7a	[ICI] + [R2] ↔ [ICIR2]	ka7a[ICI][R2] – kd7a[ICIR2]
v8	[LR2] → [pLR2]	kc8[LR2]([PKAC]/(1 + [RpcAMP]/ki_rpcamp))
v9	[pLR2] → [LR2]	kc9[pLR2]
v10	[R2] → [pR2]	kc10[R2]([PKAC]/(1 + [RpcAMP]/ki_rpcamp))
v11	[pR2] → [R2]	kc11[pR2]
v12	[ISO] + [pR2] ↔ [pLR2]	ka12[ISO][pR2] – kd12[pLR2]
G-protein activation		
v13	[Gs_GDP] → [Gs_GTP]	(kc13a[LR1] + kc13b[pLR1] + kc13c[LR2])[Gs_GDP][3)]
v14	[Gs_GTP] → [Gs_GDP]	kc14[Gs_GTP]
v15	[Gi_GDP] → [Gi_GTP]	kc15[Gi_GDP][4)][pLR2]
v16	[Gi_GTP] → [Gi_GTP]	kc16[Gi_GTP]
cAMP production		
v17	[Gs_GTP] + [AC] ↔ [GsAC]	ka17[Gs_GTP][AC][5)]/(1 + [SQ22563]/ki_22563) – kd17[GsAC]
v18	[ATP] → [cAMP]	vm18[ATP][GsAC]/(km18(1 + [Gi_GTP]/ki18) + [ATP])
v19	[cAMP] → ∅	(kc19a[PDE3] + kc19b[pp_PDE3]) [cAMP]/(km19 + [cAMP]) /(1 + [Cil]/ki_cil)
CREB activation		

v20	[cAMP] + [PKA] ↔ [PKAC]	ka20[cAMP][PKA][6] – kd20[PKAC]
v21	[CREB] → [pp_CREB]	(kc21a[PKAC]/(1 + [RpcAMP]/ki_rpcamp) + kc21b[pp_RSK])[CREB][7]/(km21 + [CREB])
v22	[pp_CREB] → [CREB]	(kc22a + kc22b[act_CN])[pp_CREB]
ICER induction		
v23	Gene → [pm_ICER]	vm23[pp_CREB]/(km23(1 + [ICER]/ki23) + [pp_CREB]) /(1 + [ICER_rnai]/ki_icerrnai)
v24	[pm_ICER] → ∅	kc24[pm_ICER]
v25	[pm_ICER] → [mm_ICER]	vm25[pm_ICER]/(km25 + [pm_ICER])
v26	[mm_ICER] → ∅	kc26[mm_ICER]
v27	[mm_ICER] → [ICER]	vm27[mm_ICER]/(km27 + [mm_ICER])
v28	[ICER] → ∅	kc28a[ICER](1 + kc28b[pp_ERK]) /(1 + ([PKAC]/(ki28(1 + [RpcAMP]/ki_rpcamp))))
PDE3 regulation		
v29	Gene → [m_PDE3]	vs29/(1 + [ICER]/ki29) + [exgpde3]/(km_exgpde3 + [exgpde3])
v30	[m_PDE3] → ∅	kc30[m_PDE3]
v31	[m_PDE3] → [PDE3]	vm31[m_PDE3]/(km31 + [m_PDE3])
v31a	[PDE3] → ∅	kc31a[PDE3]
v32	[PDE3] → [pp_PDE3]	kc32[PDE3][PKAC]/(1 + [RpcAMP]/ki_rpcamp)
v33	[pp_PDE3] → [PDE3]	kc33[pp_PDE3]
Bcl-2 production		
v34	Gene → [m_Bcl-2]	(vs34 + vm34[pp_CREB])/(km34 + [pp_CREB])/(1 + [ICER]/ki34)
v35	[m_Bcl-2] → ∅	kc35[m_Bcl-2]
v36	[m_Bcl-2] → [Bcl-2]	vm36[m_Bcl-2]/(km36 + [m_Bcl-2])
v37	[Bcl-2] → ∅	kc37[Bcl-2]
EGF-EGFR interaction		
v38	[EGF] + [EGFR] ↔ [RE]	ka38[EGF][EGFR][8] – kd38[RE]
v39	[Grb2] + [SOS] → [GS]	(ka39a[RE] + ka39b[Gi_GTP])[SOS][9][Grb2][10]/ (1 + ([pp_ERK]/ki39)^3)
v39a	[GS] → [Grb2] +[SOS]	kd39[GS]
ERK1/2 pathway		
v40	[Ras_GDP] → [Ras_GTP]	kc40[GS][Ras_GDP][11]
v41	[Ras_GTP] → [Ras_GDP]	kc41[Ras_GTP]

v42	[Raf] → [act_Raf]	kc42[Raf]$^{12)}$[Ras_GTP]
v43	[act_Raf] → [Raf]	kc43[act_Raf]
v44	[MEK] → [pp_MEK]	kc44[MEK]$^{13)}$[act_Raf]
v45	[pp_MEK] → [MEK]	kc45[pp_MEK]
v46	[ERK] → [pp_ERK]	kc46[ERK]$^{14)}$[pp_MEK]/(1 + [PD98059]/ki_pd98059)
v47	[pp_ERK] → [ERK]	kc47[pp_ERK]
v48	[RSK] → [pp_RSK]	kc48[RSK]$^{15)}$[pp_ERK]
v49	[pp_RSK] → [RSK]	kc49[pp_RSK]
Calcium regulatory machinery		
v50	[inact_CRM] → [act_CRM]	vm50a[cAMP]/(km50a + [cAMP])<br>+ vm50b([PKAC]/(1 + [RpcAMP]/ki_rpcamp))/(km50b + [PKAC])<br>+ vm50c[act_CaMKII]/((km50c + [act_CaMKII])(1 + [KN93]/ki_kn93))
v51	[act_CRM] → [inact_CRM]	kc51[act_CRM]/(km51 + [act_CRM])
v52	[CaMKII] → [act_CaMKII]	kc52[act_CRM][CaMKII]$^{16)}$/(km52 + [CaMKII])
v53	[act_CaMKII] → [CaMKII]	kc53[act_CaMKII]
v54	[CN] → [act_CN]	kc54[CN]$^{17)}$[act_CRM]
v55	[act_CN] → [CN]	kc55[act_CN]

$^{1)}$[R1] = [R1_tot] – ([pR1] + [LR1] + [pLR1] + [MetoR1]). $^{2)}$[R2] = [R2_tot] – ([pR2] + [LR2] + [pLR2] + [ICIR2]).
$^{3)}$[Gs_GDP] = [Gs_tot] – [Gs_GTP]. $^{4)}$[Gi_GDP] = [Gi_tot] – [Gi_GTP]. $^{5)}$[AC] = [AC_tot] – [GsAC].
$^{6)}$[PKA] = [PKA_tot] – [PKAC]. $^{7)}$[CREB] = [CREB_tot] – [pp_CREB]. $^{8)}$[EGFR] = [EGFR_tot] – [RE]. $^{9)}$[SOS] = [SOS_tot] – [GS]. $^{10)}$[Grb2_tot] = [Grb2]. $^{11)}$[Ras_GDP] = [Ras_tot] – [Ras_GTP]. $^{12)}$[Raf] = [Raf_tot] – [act_Raf]. $^{13)}$[MEK] = [MEK_tot] – [pp_MEK]. $^{14)}$[ERK] = [ERK_tot] – [pp_ERK]. $^{15)}$[RSK] = [RSK_tot] – [pp_RSK]. $^{16)}$[CaMKII] = [CaMKII_tot] – [act_CaMKII]. $^{17)}$[CN]=[CN_tot] – [act_CN].

Supplementary Table 2. Ordinary differential equations (ODEs)

$\frac{d[LR1]}{dt} = v1 - v2 + v3$	(1)	$\frac{d[pLR1]}{dt} = v2 - v3 + v6$	(2)	$\frac{d[pR1]}{dt} = v4 - v5 - v6$	(3)
$\frac{d[MetoR1]}{dt} = v1a$	(4)	$\frac{d[LR2]}{dt} = v7 - v8 + v9$	(5)	$\frac{d[pLR2]}{dt} = v8 - v9 + v12$	(6)
$\frac{d[pR2]}{dt} = v10 - v11 - v12$	(7)	$\frac{d[ICIR2]}{dt} = v7a$	(8)	$\frac{d[Gs\_GTP]}{dt} = v13 - v14$	(9)
$\frac{d[Gi\_GTP]}{dt} = v15 - v16$	(10)	$\frac{d[ACGs]}{dt} = v17$	(11)	$\frac{d[cAMP]}{dt} = v18 - v19$	(12)
$\frac{d[PKAC]}{dt} = v20$	(13)	$\frac{d[pp\_CREB]}{dt} = v21 - v22$	(14)	$\frac{d[pm\_ICER]}{dt} = v23 - v24$	(15)
$\frac{d[mm\_ICER]}{dt} = v25 - v26$	(16)	$\frac{d[ICER]}{dt} = v27 - v28$	(17)	$\frac{d[mm\_PDE3]}{dt} = v29 - v30$	(18)
$\frac{d[PDE3]}{dt} = \begin{cases} v31 - v31a - v32 \\ +v33 \end{cases}$	(19)	$\frac{d[pp\_PDE3]}{dt} = v32 - v33$	(20)	$\frac{d[mm\_Bcl2]}{dt} = v34 - v35$	(21)
$\frac{d[Bcl2]}{dt} = v36 - v37$	(22)	$\frac{d[RE]}{dt} = v38$	(23)	$\frac{d[GS]}{dt} = v39$	(24)
$\frac{d[Ras\_GTP]}{dt} = v40 - v41$	(25)	$\frac{d[act\_Raf]}{dt} = v42 - v43$	(26)	$\frac{d[pp\_MEK]}{dt} = v44 - v45$	(27)
$\frac{d[pp\_ERK]}{dt} = v46 - v47$	(28)	$\frac{d[pp\_RSK]}{dt} = v48 - v49$	(29)	$\frac{d[act\_CRM]}{dt} = v50 - v51$	(30)
$\frac{d[act\_CaMKII]}{dt} = v52 - v53$	(31)	$\frac{d[act\_CN]}{dt} = v54 - v55$	(32)		

Supplementary Table 3. State variables

State variable	Initial Value [μM]	State variable	Initial Value [μM]	State variable	Initial Value [μM]
[*LR1*]	0.000E+00	[*pLR1*]	0.000E+00	[*pR1*]	0.000E+00
[*MetoR1*]	0.000E+00	[*LR2*]	0.000E+00	[*pLR2*]	0.000E+00
[*pR2*]	0.000E+00	[*ICIR2*]	0.000E+00	[*Gs_GTP*]	6.494E–03
[*Gi_GTP*]	0.000E+00	[*ACGs*]	0.000E+00	[*cAMP*]	0.000E+00
[*PKAC*]	0.000E+00	[*pp_CREB*]	0.000E+00	[*pm_ICER*]	0.000E+00
[*mm_ICER*]	0.000E+00	[*ICER*]	0.000E+00	[*mm_PDE3*]	4.899E–01
[*PDE3*]	6.494E–03	[*pp_PDE3*]	0.000E+00	[*mm_Bcl2*]	1.013E–01
[*Bcl2*]	3.331E–06	[*RE*]	0.000E+00	[*GS*]	0.000E+00
[*Ras_GTP*]	0.000E+00	[*act_Raf*]	0.000E+00	[*pp_MEK*]	0.000E+00
[*pp_ERK*]	0.000E+00	[*pp_RSK*]	0.000E+00	[*act_CRM*]	0.000E+00
[*act_CaMKII*]	0.000E+00	[*act_CN*]	0.000E+00		

To determine the initial values for state variables, we calculated the steady-state values of all state variables in the absence of receptor stimulation and then used them as the initial values of the model for any other simulation study.

Supplementary Table 4. Kinetic parameters

Parameter	Value	Unit	Ref.	Parameter	Value	Unit	Ref.
ka1	1.429E+02	$(\mu M)^{-1}\cdot min^{-1}$	estimated	*kd1*	2.151E+01	min^{-1}	estimated
ka1a	9.906E+01	$(\mu M)^{-1}\cdot min^{-1}$	estimated	*kd1a*	9.906E–01	min^{-1}	estimated
kc2	2.453E+01	$(\mu M)^{-1}\cdot min^{-1}$	estimated	*kc3*	1.677E–01	min^{-1}	estimated
kc4	6.014E+03	$(\mu M)^{-1}\cdot min^{-1}$	estimated	*kc5*	2360E–03	min^{-1}	estimated
ka6	4.324E–02	$(\mu M)^{-1}\cdot min^{-1}$	estimated	*kd6*	1.134E+02	min^{-1}	estimated
ka7	1.159E+02	$(\mu M)^{-1}\cdot min^{-1}$	estimated	*kd7*	3.689E+01	min^{-1}	estimated
ka7a	9.900E+01	$(\mu M)^{-1}\cdot min^{-1}$	estimated	*kd7a*	6.237E+01	min^{-1}	estimated
kc8	9.825E–01	$(\mu M)^{-1}\cdot min^{-1}$	estimated	*kc9*	2.147E–01	min^{-1}	estimated
kc10	3.892E+02	$(\mu M)^{-1}\cdot min^{-1}$	estimated	*kc11*	2.808E+02	min^{-1}	estimated
ka12	4.116E+01	$(\mu M)^{-1}\cdot min^{-1}$	estimated	*kd12*	3.969E+00	min^{-1}	estimated
kc13a	2.254E+01	$(\mu M)^{-1}\cdot min^{-1}$	estimated	*kc13b*	5.710E–02	$(\mu M)^{-1}\cdot min^{-1}$	estimated
kc13c	5.312E+01	$(\mu M)^{-1}\cdot min^{-1}$	estimated	*kc14*	1.538E+00	min^{-1}	estimated
kc15	4.428E+02	$(\mu M)^{-1}\cdot min^{-1}$	estimated	*kc16*	1.520E+00	min^{-1}	estimated
ka17	1.944E+02	$(\mu M)^{-1}\cdot min^{-1}$	estimated	*kd17*	2.100E+01	min^{-1}	estimated
vm18	2.248E+01	min^{-1}	estimated	*km18*	4.557E+00	mM	estimated
ki18	5.884E–03	μM	estimated	*kc19a*	2.216E+02	min^{-1}	estimated
kc19b	9.102E+00	min^{-1}	estimated	*km19*	3.533E–02	μM	estimated
ka20	5.925E+00	$(\mu M)^{-1}\cdot min^{-1}$	estimated	*kd20*	2.887E+00	min^{-1}	estimated
kc21a	7.671E+01	min^{-1}	estimated	*kc21b*	2.396E+00	min^{-1}	estimated
km21	2.500E–02	μM	estimated	*kc22a*	3.109E–01	min^{-1}	estimated
kc22b	2.276E+01	$(\mu M)^{-1}\cdot min^{-1}$	estimated	*vm23*	2.639E+00	$\mu M\cdot min^{-1}$	estimated
km23	1.126E+01	μM	estimated	*ki23*	2.263E–01	μM	estimated
kc24	3.507E–02	min^{-1}	estimated	*vm25*	4.493E+00	$\mu M\cdot min^{-1}$	estimated
km25	5.460E+01	μM	estimated	*kc26*	2.759E–01	min^{-1}	estimated
vm27	1.353E+00	$\mu M\cdot min^{-1}$	estimated	*km27*	2.390E+01	μM	estimated
kc28a	1.473E–03	min^{-1}	estimated	*kc28b*	2.041E+03	$(\mu M)^{-1}$	estimated
ki28	4.320E–05	μM	estimated	*vs29*	2.300E+00	$\mu M\cdot min^{-1}$	estimated

ki29	1.125E–01	μM	estimated	*kc30*	4.694E+00	min^{-1}	estimated
vm31	4.304E+00	$\mu M \cdot min^{-1}$	estimated	*km31*	2.124E+01	μM	estimated
kc31a	1.494E+01	min^{-1}	estimated	*kc32*	3.010E+02	$(\mu M)^{-1} \cdot min^{-1}$	estimated
kc33	4.926E+00	min^{-1}	estimated	*vs34*	4.633E–03	$(\mu M)^{2} \cdot min^{-1}$	estimated
vm34	9.916E–01	$\mu M \cdot min^{-1}$	estimated	*km34*	1.149E–01	μM	estimated
ki34	5.729E–01	μM	estimated	*kc35*	3.978E–01	min^{-1}	estimated
vm36	8.863E–01	$\mu M \cdot min^{-1}$	estimated	*km36*	1.001E+02	μM	estimated
kc37	1.953E–02	min^{-1}	estimated	*ka38*	3.360E+01	$(\mu M)^{-1} \cdot min^{-1}$	estimated
kd38	6.925E+01	min^{-1}	estimated	*ka39a*	1.088E+05	$(\mu M)^{-2} \cdot min^{-1}$	estimated
ka39b	5.714E+03	$(\mu M)^{-2} \cdot min^{-1}$	estimated	*ki39*	7.716E–04	μM	estimated
kd39	1.616E-01	min^{-1}	estimated	*kc40*	4.869E+02	$(\mu M)^{-1} \cdot min^{-1}$	estimated
kc41	5.073E+01	min^{-1}	estimated	*kc42*	4.648E+02	$(\mu M)^{-1} \cdot min^{-1}$	estimated
kc43	4.663E+00	min^{-1}	estimated	*kc44*	4.037E+02	$(\mu M)^{-1} \cdot min^{-1}$	estimated
kc45	6.872E–02	min^{-1}	estimated	*kc46*	7.821E+00	$(\mu M)^{-1} \cdot min^{-1}$	estimated
kc47	3.905E–01	min^{-1}	estimated	*kc48*	2.970E–01	$(\mu M)^{-1} \cdot min^{-1}$	estimated
kc49	3.454E+00	min^{-1}	estimated	*vm50a*	1.892E–01	$\mu M \cdot min^{-1}$	estimated
km50a	3.980E–05	μM	estimated	*vm50b*	3.961E+00	$\mu M \cdot min^{-1}$	estimated
km50b	1.166E+01	μM	estimated	*vm50c*	4.230E–01	$\mu M \cdot min^{-1}$	estimated
km50c	8.802E–01	μM	estimated	*kc51*	3.837E+00	$\mu M \cdot min^{-1}$	estimated
km51	9.000E+01	μM	estimated	*kc52*	8.300E–05	min^{-1}	estimated
km52	5.395E–02	μM	estimated	*kc53*	7.939E+00	min^{-1}	estimated
kc54	2.306E–02	$(\mu M)^{-1} \cdot min^{-1}$	estimated	*kc55*	1.564E+01	min^{-1}	estimated
ki_rpcamp	1.500E+04	μM	estimated	*ki_cil*	2.000E+01	μM	estimated
ki_icerrnai	3.000E+01	μM	estimated	*ki_chx*	5.000E+02	μM	estimated
ki_pd98059	3.000E+02	μM	estimated	*ki_kn93*	1.000E+01	μM	estimated
ki_SQ2256	2.000E+01	μM	estimated	*km_exgpde3*	2.000E+01	μM	estimated

Supplementary Table 5. Constant values

Species	Value	Unit	Ref.	Species	Value	Unit	Ref.
[*R1_tot*]	2.760E-02	μM	estimated	[*R2_tot*]	6.410E-03	μM	estimated
[*Gs_tot*]	3.570E-01	μM	estimated	[*Gi_tot*]	3.930E-01	μM	estimated
[*AC_tot*]	1.590E-01	μM	estimated	[*ATP*]	5.000E+00	μM	estimated
[*PKA_tot*]	6.930E-03	μM	estimated	[*CREB_tot*]	4.220E-02	μM	estimated
[*EGFR_tot*]	1.990E-03	μM	estimated	[*Grb2_tot*]	7.740E-02	μM	estimated
[*SOS_tot*]	1.180E-02	μM	estimated	[*Ras_tot*]	1.250E-01	μM	estimated
[*Raf_tot*]	2.170E-01	μM	estimated	[*MEK_tot*]	6.740E-02	μM	estimated
[*ERK_tot*]	2.260E-01	μM	estimated	[*RSK_tot*]	7.160E-02	μM	estimated
[*CaMKII_tot*]	7.060E-02	μM	estimated	[*CN_tot*]	1.660E-01	μM	estimated

We assumed that the volume of cardiomyocytes is 26.1 pL (where the radius of cardiomyocyte is 8 μm and the length is 130 μm) and the protein amount in one cardiomyocyte is 2.8 ng (where the number of cells in a 35-mm culture dish is 95,000 and the total amount of protein from one 35-mm culture dish is 270 μg). Based on these parameter values, the total concentration of the protein was estimated from the RNA-seq data [1] using the following equation:

$$Conc(M) = \left(\sum_{j=1}^{M} RPKM_j \Big/ \sum_{i=1}^{N} RPKM_i \right) \cdot \left(1/MW \right) \cdot \left(A/V \right)$$

where $RPKM$ is the Reads Per Kilobase per Million mapped reads, $Conc$ is the concentration of a protein, M is the total number of isoforms of the protein, N is the number of genes, MW is the molecular weight of the protein, A is the protein amount of the cardiomyocyte (2.8 ng), and V is the volume of the cardiomyocyte (26.1 pL). We also assumed that the amount of a protein is linearly correlated with the amount of mRNA [2].

Supplementary Table 6. Potential therapeutic targets.

Target	Drug name	Kinetic parameter	Chemical name	Description	Reference
G_s	NF499	*kc13a*, *kc13b*, *kc13c*	4,4′,4",4′"-[carbonyl-bis[imino-5,1,3-benzenetriyl bis-(carbonylimino)]]tetrakis-benzene-1,3-disulfonate	Inhibition of GTP binding to $G_s\alpha$	3
	NF503		4,4′-[carbonylbis[imino-3,1-phenylene-(2,5-benzimidazolylene)carbonylimino]]bis-benzenesulfonate		
G_i	GOT	*kc15*	guanosine 5′-O-(2-thiodiphosphate)	GDP analog which inhibits G_i	4
Grb2	CGP78850	*ka41a*, *ka41b*	peptide	Selective, potent inhibitor of Grb2 SH2 domain	5
AC	BPIPP	*vm18*	5-(3-Bromophenyl)-5,11-dihydro-1,3-dimethyl-1H-indeno[2',1':5,6]pyrido[2,3-d]pyrimidine-2,4,6(3H)-trione	Non-competitive adenylyl cyclase inhibitor	6
	KH7		(±)-2-(1H-benzimidazol-2-ylthio)propanoic acid 2-[(5-bromo-2-hydroxyphenyl)methylene]hydrazide	Selective soluble adenylyl cyclase inhibitor	7
	SQ22536		9-(Tetrahydro-2-furanyl)-9H-purin-6-amine	Inhibitor of adenylyl cyclase	8
cAMP	Rp-cAMPS	*ka20*	(R)-Adenosine, cyclic 3',5'-(hydrogenphosphorothioate) triethylammonium	Competitive inhibitor of cAMP-induced activation of PKA by interacting with cAMP binding sites on the regulatory subunit	9, 10
	8-Bromo-cAMP		8-Bromoadenosine-3',5'-cyclic monophosphate sodium salt		
	Dibutyryl-cAMP		N6,O2'-Dibutyryl adenosine 3',5'-cyclic monophosphate sodium salt		

PDE3	Anagrelide	*kc19a*, *kc19b*	6,7-Dichloro-1,5-dihydroimidazo[2,1-b]quinazolin-2(3H)-one hydrochloride	Potent PDE3 inhibitor	11
	Cilostamide		N-Cyclohexyl-N-methyl-4-(1,2-dihydro-2-oxo-6-quinolyloxy)butyramide	Selective PDE3 inhibitor	12
	Cilostazol		6-[4-(1-Cyclohexyl-1H-tetrazol-5-yl)butoxy]-3,4-dihydro-2(1H)-quinolinone	Potent PDE3A inhibitor	13
	Enoximone		1,3-Dihydro-4-methyl-5-[4-(methylthio)benzoyl]-2H-imidazol-2-one	PDE3 inhibitor	14
	Milrinone		1,6-Dihydro-2-methyl-6-oxo-(3,4'-bipyridine)-5-carbonitrile	Potent PDE3 inhibitor	15
	Siguazodan		N-Cyano-N'-methyl-N''-[4-(1,4,5,6-tetrahydro-4-methyl-6-oxo-3-pyridazinyl)phenyl]guanidine	Selective PDE3 inhibitor	
Ras	Salirasib	*kc42*, *kc43*	S-trans, trans-farnesylthiosalicylic acid	Ras inhibitor that dislodges all active Ras isoforms from the membrane	16
Raf	GDC0879	*kc46*	(E)-2,3-Dihydro-5-[1-(2-hydroxyethyl)-3-(4-pyridinyl)-1H-pyrazol-4-yl]-1H-inden-1-one oxime	Potent and selective b-Raf inhibitor	17
	GW5074		3-(3,5-Dibromo-4-hydroxy-benzylidene)-5-iodo-1,3-dihydro-indol-2-one	Potent, selective c-Raf inhibitor	18
	L-779,450		2-Chloro-5-[2-Phenyl-5-(4-pyridinyl)-1H-imidazol-4-yl]phenol	Potent, ATP-competitive Raf inhibitor	19

	SB590885		5-[2-[4-[2-(Dimethylamino)ethoxy]phenyl]-5-(4-pyridinyl)-1H-imidazol-4-yl]-2,3-dihydro-1H-inden-1-one oxime	Potent, selective b-Raf inhibitor	
MEK	PD0325901	*kc48*	N-[(2R)-2,3-Dihydroxypropoxy]-3,4-difluoro-2-[(2-fluoro-4-iodophenyl)amino]-benzamide	Selective inhibitor of MEK1/2	20
	PD184352		2-[(2-Chloro-4-iodophenyl)amino]-N-cyclopropylmethoxy)-3,4-difluorobenzamide		21
	U0126		1,4-Diamino-2,3-dicyano-1,4-bis[2-aminophenylthio]butadiene		22
	PD198306		N-(Cyclopropylmethoxy)-3,4,5-trifluoro-2-[(4-iodo-2-methylphenyl)amino]-benzamide		23
	PD98059	*kc46*	2-(2-Amino-3-methoxyphenyl)-4H-1-benzopyran-4-one	Binds to the inactivated MEK, preventing its phosphorylation by c-Raf or MEK kinase	24
ERK1/2	FR180204	*kc50*	5-(2-Phenyl-pyrazolo[1,5-a]pyridin-3-yl)-1H-pyrazolo[3,4-c]pyridazin-3-ylamine	Selective ERK1/2 inhibitor	25

RSK	BRD7389	*kc21b*	1-[(2-Phenylethyl)amino]-3H-naphtho[1,2,3-de]quinoline-2,7-dione	RSK inhibitor	26
	PF4708671		2-[[4-(5-Ethylpyrimidin-4-yl)piperazin-1-yl]methyl]-5-(trifluoromethyl)-1H-benzo[d]imidazole		27
	SL0101-1		3-[(3,4-Di-O-acetyl-6-deoxy-α-L-mannopyranosyl)oxy]-5,7-dihydro-2-(4-hydroxyphenyl)-4H-1benzopyran-4-one		28
PKA	KT5720	*kc21a*	(9R,10S,12S)-2,3,9,10,11,12-Hexahydro-10-hydroxy-9-methyl-1-oxo-9,12-epoxy-1H-diindolo[1,2,3-fg:3',2',1'-kl]pyrrolo[3,4-i][1,6]benzodiazocine-10-carboxylic acid, hexyl ester	Potent, selective PKA inhibitor	29
	PKA inhibitor fragment(6-22) amide		peptide	Potent PKA inhibitor	30
	PKI 14-22 amide, myristoylated			Permeable PKA inhibitor	31
CaMKII	Autocamtide-2-related inhibitory peptide	*vm52c*	peptide	Potent, selective CaMKII inhibitor	32

	KN-62	*kc54*	4-[(2S)-2-[(5-isoquinolinylsulfonyl)methylamino]-3-oxo-3-(4-phenyl-1-piperazinyl)propyl] phenyl isoquinolinesulfonic acid ester	Selective, permeable CaMKII inhibitor	33
	KN-93	*kc54*	N-[2-[[[3-(4-Chlorophenyl)-2-propenyl]methylamino]methyl]phenyl]-N-(2-hydroxyethyl)-4-methoxybenzenesulphonamide	Potent, permeable CaMKII inhibitor	34
ICER	siRNA	*kc24*, *kc26*	RNA sequences	Accelerates degradation of ICER mRNA	35

III. Supplementary Notes

Supplementary Note 1. Mathematical model

The computational model of β-AR signalling network is composed of four major modules: cAMP-PKA signalling module, central feedback regulation module, extracellular signal-regulated kinases 1 and 2 (ERK1/2) signalling module and Ca^{2+} regulatory module. The cAMP-PKA signalling module includes two types of β-AR (β_1 and β_2) which sequentially activate G_s protein and adenylyl cyclase (AC) [36, 37]. The active AC promotes the accumulation of cytosolic cAMP and subsequently leads to PKA activation [38]. The active PKA phosphorylates β_1- and β_2-AR simultaneously; however, the functional consequences of these two receptors are quite different [37, 39, 40]: The phosphorylated β_1-AR is internalized and thus desensitized to the stimulation of agonist, while the phosphorylated β_2-AR is coupled from G_s to G_i [41]. Similar to PKA, GRK2 also phosphorylates both types of β-ARs but the difference is that GRK2 phosphorylates only the ligand bound receptors [42, 43]. Thus, the beta-AR signalling pathway is regulated by two feedback loops that are mediated by PKA and GRK2. In this study, for the simplicity of mathematical modeling, we explicitly included only the feedback loops of PKA in the model, since we assumed that the feedback mechanism mediated through PKA and GRK2 are functionally redundant in terms of the cell fate determination. Note, however, that the effect of GRK2 was still reflected in our model because the model parameters were fitted to the time course data, where the functions of GRK2 were already reflected.

The central feedback regulation module consists of CREB, ICER and PDE3. The active PKA is translocated to the nucleus and phosphorylates CREB, which leads to the transcription of inducible cAMP early repressor (ICER) and Bcl-2 [44]. The induced ICER protein is further stabilized by cAMP-dependent signalling [45]. ICER protein contains DNA binding and leucine zipper domains but not the N-terminal transactivation domain, which makes it function as endogenous inhibitors of gene transcription driven by its cognates such as CREB, including its own expression by competing with CREB in binding CRE sequence [44]. In addition, ICER transcriptionally represses the expression of PDE3A, which subsequently contributes to the acceleration of the cAMP accumulation [44]. Thus, in modeling, ICER mediates two feedback loops: one is constructed by its own negative autoregulation and the other one is PDE3-mediated feedback loop. Note that although a number of experimental results showed that PDE4 may play an essential role in cellular processes such as muscle contraction [46], for the simplicity of mathematical modeling, we explicitly included only PDE3 in the model since PDE4 does not significantly affect the apoptosis of cardiomyocytes, even though its inhibition increases the cAMP level [47]. However, the functional effect of PDE4 was implicitly reflected in our model by fitting the model parameters to the time-course data of cAMP and PKA measured in the presence of PDE4. Regarding the compartmentalization of cAMP signal, we also simplified the model by assuming that the cAMP signal in cytosol is homogeneous, since the coarse-grained network that simplifies intermediate signalling steps would not significantly affect the steady-state properties of the system [48].

As mentioned above, PKA switches the coupling of β_2-AR for G_s to G_i. This switching releases G_i_α_GTP from G_i_β/γ subunit. G_i_α_GTP inhibits the AC activity while promoting the binding of SOS and Grb2 via β-arrestins, which subsequently activates the ERK1/2 signalling module [41]. ERK1/2 regulates the central feedback module in two ways: the active ERK1/2 destabilizes the ICER protein [45] and activates CREB via the phosphorylation of RSK [49, 50]. The Ca^{2+} regulatory module is quite complex because it includes many channels and pumps that regulate the inward and outward Ca^{2+} flux through the plasma membrane and intracellular Ca^{2+} store [51]. However, we do not intend to investigate all of the detailed mechanism in this study. Instead, we construct a basic and minimal module. As a result, this module includes the Ca^{2+} regulatory machinery, calcineurin and Ca^{2+}/calmodulin-dependent protein kinase II, which regulates the β-AR signalling network through feedforward and feedback regulations.

The model was developed using ordinary differential equations (ODEs) on the basis of Michaelis-Mention-type functions and mass action law. In particular, the reaction steps of β_1- and β_2-ARs in the model are completed in a closed system, where all components are linked by reversible reactions and thereby the reactions consist of a closed-loop. Thus, for these reaction steps we applied detailed balance principle as previously reported [52, 53]. In other words, the product of rate constants in one direction around the closed loop is equal to the product of rate constants in the opposite direction around the loop. As explained in the above, the simplification of some signalling components might reduce the accuracy of simulated temporal dynamics. However, that would not much significantly affect the long-term response in terms of cell fate determination [48].

Supplementary Note 2. Simplified ODE model

$$\frac{d[PKA]}{dt} = V_s \frac{[ISO]^{ns}}{K_s^{\ ns} + [ISO]^{ns}} \frac{K_{i7}^{\ ni7}}{K_{i7}^{\ ni7} + [PDE]^{ni7}} - \frac{1}{\tau_{PKA}}[PKA],$$

$$\frac{d[Bcl2]}{dt} = \left(V_{Bcl2} + V_{11} \frac{[ERK]^{n11}}{K_{11}^{n11} + [ERK]^{n11}} \right) \frac{K_{i5}^{\ ni5}}{K_{i5}^{\ ni5} + [ICER]^{ni5}} - \frac{1}{\tau_{Bcl2}}[Bcl2],$$

$$\frac{d[PDE]}{dt} = V_{PDE} \frac{K_{i4}^{\ ni4}}{K_{i4}^{\ ni4} + [ICER]^{ni4}} - \frac{1}{\tau_{PDE}}[PDE],$$

$$\frac{d[ICER]}{dt} = \left(V_2 \frac{[Bcl2]^{n2}}{K_2^{\ n2} + [Bcl2]^{n2}} + V_{15} \frac{[PKA]^{n15}}{K_{15}^{\ n15} + [PKA]^{n15}} \right) \frac{K_{i10}^{\ ni10}}{K_{i10}^{\ ni10} + [ERK]^{ni10}} - \frac{1}{\tau_{ICER}}[ICER],$$

$$\frac{d[ERK]}{dt} = V_{ERK} \frac{[PKA]^{n9}}{K_9^{\ n9} + [PKA]^{n9}} - \frac{1}{\tau_{ERK}}[ERK].$$

Supplementary Note 3. Sensitivity analysis

We carried out the global sensitivity analysis by perturbing the kinetic parameter values of all the regulatory links (39 links in total) in the range of 10-fold, and examined their influences on the Bcl-2 switching response profile. The results of this global sensitivity analysis showed that the parameters (*vm18*, *kc19a*, *ki39*, *kc48*, *kc21b*, *vm23*, *vm25*, *vm27*, *vm34* and *ki34*) associated with eight essential regulatory links have significant effects on shaping the Bcl-2 switching response profile (Supplementary Fig. 12a), consistent with the results obtained from our coarse-graining network analysis (Fig. 5). In the next, we further investigated the hidden mechanism underlying the cellular decision for survival or death response of Bcl-2. For this purpose, the parameter sets were clustered into two groups leading to either 'survival-only' or 'death-only' response which was defined on the basis of Bcl-2 response profile. By comparing the clustered parameter values, we found that most of the parameters (e.g., *kc19b*, *kc19a*, *kc28b*, *ka39b*, *vm23*, *ki23*, *ki29* and *ki34*) representing the eight essential regulatory links (Fig. 5b) showed statistically significant differences, implying that they play a central role in determining the Bcl-2 switching response property (Supplementary Fig. 12b).

Supplementary Note 4. Nondimensionalized ODE equations

$$\frac{d[PKA]}{dt}=\frac{1}{\tau_{PKA}}\left(\frac{[ISO]^{ns}}{K_s{}^{ns}+[ISO]^{ns}}\frac{K_{i7}{}^{ni7}}{K_{i7}{}^{ni7}+[PDE]^{ni7}}-[PKA]\right),$$

$$\frac{d[Bcl2]}{dt}=\frac{1}{\tau_{Bcl2}}\left(\left(1+\beta_{11}\frac{[ERK]^{n11}}{K_{11}^{n11}+[ERK]^{n11}}\right)\cdot\frac{K_{i5}{}^{ni5}}{K_{i5}{}^{ni5}+[ICER]^{ni5}}-[Bcl2]\right),$$

$$\frac{d[PDE]}{dt}=\frac{1}{\tau_{PDE}}\left(\frac{K_{i4}{}^{ni4}}{K_{i4}{}^{ni4}+[ICER]^{ni4}}-[PDE]\right),$$

$$\frac{d[ICER]}{dt}=\frac{1}{\tau_{ICER}}\left(\left(\frac{[Bcl2]^{n2}}{K_2{}^{n2}+[Bcl2]^{n2}}+\beta_{15}\frac{[PKA]^{n15}}{K_{15}{}^{n15}+[PKA]^{n15}}\right)\cdot\frac{K_{i10}{}^{ni10}}{K_{i10}{}^{ni10}+[ERK]^{ni10}}-[ICER]\right),$$

$$\frac{d[ERK]}{dt}=\frac{1}{\tau_{ERK}}\left(\frac{[PKA]^{n9}}{K_9{}^{n9}+[PKA]^{n9}}-[ERK]\right),$$

where $\frac{[PKA]}{V_s\tau_{PKA}}\Rightarrow[PKA]$, $\frac{[Bcl2]}{V_{Bcl2}\tau_{Bcl2}}\Rightarrow[Bcl2]$, $\frac{[PDE]}{V_{PDE}\tau_{PDE}}\Rightarrow[PDE]$, $\frac{[ICER]}{V_2\tau_{ICER}}\Rightarrow[ICER]$, $\frac{[ERK]}{V_{ERK}\tau_{ERK}}\Rightarrow[ERK]$, $\frac{K_{i7}}{V_{PDE}\tau_{PDE}}\Rightarrow K_{i7}$, $\frac{K_{i5}}{V_2\tau_{IC}}\Rightarrow K_{i5}$, $\frac{K_{i11}}{V_{ERK}\tau_{ERK}}\Rightarrow K_{i11}$, $\frac{K_{i4}}{V_2\tau_{IC}}\Rightarrow K_{i4}$, $\frac{K_{15}}{V_s\tau_{PKA}}\Rightarrow K_{15}$, $\frac{K_2}{V_{Bcl2}\tau_{Bcl2}}\Rightarrow K_2$, $\frac{K_{i10}}{V_{ERK}\tau_{ERK}}\Rightarrow K_{i10}$, $\frac{K_9}{V_s\tau_{PKA}}\Rightarrow K_9$, $\frac{V_{15}}{V_2}\Rightarrow\beta_{15}$, $\frac{V_{11}}{V_{Bcl2}}\Rightarrow\beta_{11}$.

IV. Supplementary References

1. Song HK, Hong SE, Kim T, Kim DH. Deep RNA sequencing reveals novel cardiac transcriptomic signatures for physiological and pathological hypertrophy. *PLoS One* **7**, e35552 (2012).
2. Nie L, Wu G, Culley DE, Scholten JC, Zhang W. Integrative analysis of transcriptomic and proteomic data: challenges, solutions and applications. *Crit Rev Biotechnol* **27**, 63-75 (2007).
3. Hohenegger M*, et al.* Gsα-selective G protein antagonists. *Proc Natl Acad Sci U S A* **95**, 346-351 (1998).
4. Moon EY, Kim HS, Im YS. Gi-protein inhibitor, guanosine 5'-O-(2-thiodiphosphate), induces senescence-associated beta-galactosidase positive cell formation through CREB phosphorylation. *Life sciences* **86**, 683-690 (2010).
5. Gay B*, et al.* Effect of potent and selective inhibitors of the Grb2 SH2 domain on cell motility. *J Biol Chem* **274**, 23311-23315 (1999).
6. Kots AY*, et al.* Pyridopyrimidine derivatives as inhibitors of cyclic nucleotide synthesis: Application for treatment of diarrhea. *Proc Natl Acad Sci U S A* **105**, 8440-8445 (2008).
7. Kumar S, Kostin S, Flacke JP, Reusch HP, Ladilov Y. Soluble adenylyl cyclase controls mitochondria-dependent apoptosis in coronary endothelial cells. *J Biol Chem* **284**, 14760-14768 (2009).
8. Harris D, Asaad M, Phillips M, Goldenberg H, Antonaccio M. Inhibition of adenylate cyclase in human blood platelets by 9-substituted adenine derivatives. *J Cyclic Nucleotide Res* **5**, 125 (1979).

9. Van Haastert P, Van Driel R, Jastorff B, Baraniak J, Stec W, De Wit R. Competitive cAMP antagonists for cAMP-receptor proteins. *J Biol Chem* **259**, 10020-10024 (1984).

10. Hei YJ, MacDonell KL, McNeill JH, Diamond J. Lack of correlation between activation of cyclic AMP-dependent protein kinase and inhibition of contraction of rat vas deferens by cyclic AMP analogs. *Mol Pharmacol* **39**, 233-238 (1991).

11. Gillespie E. Anagrelide: a potent and selective inhibitor of platelet cyclic AMP phosphodiesterase enzyme activity. *Biochem Pharmacol* **37**, 2866 (1988).

12. Hidaka H*, et al.* Selective inhibitor of platelet cyclic adenosine monophosphate phosphodiesterase, cilostamide, inhibits platelet aggregation. *J Pharmacol Exp Ther* **211**, 26-30 (1979).

13. Shintani S*, et al.* General pharmacological properties of cilostazol, a new antithrombotic drug. Part II: Effect on the peripheral organs. *Arzneimittel-Forschung* **35**, 1163 (1985).

14. Pacher R, Stanek B. Ambulatory vasodilator therapy in heart failure: systematic review of the literature and personal observational experience. *Eur J Heart Fail* **1**, 263-268 (1999).

15. Mary Tang K, Jang EK, Haslam RJ. Photoaffinity labelling of cyclic GMP-inhibited phosphodiesterase (PDE III) in human and rat platelets and rat tissues: effects of phosphodiesterase inhibitors. *Eur J Pharmacol* **268**, 105-114 (1994).

16. Barkan B, Starinsky S, Friedman E, Stein R, Kloog Y. The Ras inhibitor farnesylthiosalicylic acid as a potential therapy for neurofibromatosis type 1. *Clin Cancer Res* **12**, 5533-5542 (2006).

17. Hoeflich KP*, et al.* Antitumor efficacy of the novel RAF inhibitor GDC-0879 is

predicted by BRAFV600E mutational status and sustained extracellular signal-regulated kinase/mitogen-activated protein kinase pathway suppression. *Cancer Res* **69**, 3042-3051 (2009).

18. Lackey K*, et al.* The discovery of potent cRaf1 kinase inhibitors. *Bioorg Med Chem Lett* **10**, 223-226 (2000).

19. Takle AK*, et al.* The identification of potent and selective imidazole-based inhibitors of B-Raf kinase. *Bioorg Med Chem Lett* **16**, 378-381 (2006).

20. Sebolt-Leopold JS*, et al.* The biological profile of PD 0325901: a second generation analog of CI-1040 with improved pharmaceutical potential. *Proc Am Assoc Cancer Res* **2004**, 925 (2004).

21. Nguyen TK, Rahmani M, Harada H, Dent P, Grant S. MEK1/2 inhibitors sensitize Bcr/Abl+ human leukemia cells to the dual Abl/Src inhibitor BMS-354/825. *Blood* **109**, 4006-4015 (2007).

22. Duncia JV*, et al.* MEK inhibitors: the chemistry and biological activity of U0126, its analogs, and cyclization products. *Bioorg Med Chem Lett* **8**, 2839-2844 (1998).

23. Ciruela A, Dixon A, Bramwell S, Gonzalez M, Pinnock R, Lee K. Identification of MEK1 as a novel target for the treatment of neuropathic pain. *Br J Pharmacol* **138**, 751-756 (2009).

24. Alessi DR, Cuenda A, Cohen P, Dudley DT, Saltiel AR. PD 098059 is a specific inhibitor of the activation of mitogen-activated protein kinase kinase in vitro and in vivo. *J Biol Chem* **270**, 27489-27494 (1995).

25. Ohori M*, et al.* Identification of a selective ERK inhibitor and structural determination of the inhibitor–ERK2 complex. *Biochem Biophys Res Commun*

336, 357-363 (2005).

26. Fomina-Yadlin D*, et al.* Small-molecule inducers of insulin expression in pancreatic α-cells. *Proc Natl Acad Sci U S A* **107**, 15099-15104 (2010).

27. Pearce LR*, et al.* Characterization of PF-4708671, a novel and highly specific inhibitor of p70 ribosomal S6 kinase (S6K1). *The Biochemical journal* **431**, 245-255 (2010).

28. Smith JA, Poteet-Smith CE, Xu Y, Errington TM, Hecht SM, Lannigan DA. Identification of the first specific inhibitor of p90 ribosomal S6 kinase (RSK) reveals an unexpected role for RSK in cancer cell proliferation. *Cancer Res* **65**, 1027-1034 (2005).

29. Gadbois DM, Crissman HA, Tobey RA, Bradbury EM. Multiple kinase arrest points in the G1 phase of nontransformed mammalian cells are absent in transformed cells. *Proc Natl Acad Sci U S A* **89**, 8626-8630 (1992).

30. Glass DB, Lundquist LJ, Katz BM, Walsh DA. Protein kinase inhibitor-(6-22)-amide peptide analogs with standard and nonstandard amino acid substitutions for phenylalanine 10. Inhibition of cAMP-dependent protein kinase. *J Biol Chem* **264**, 14579-14584 (1989).

31. Glass DB, Cheng HC, Mende-Mueller L, Reed J, Walsh DA. Primary structural determinants essential for potent inhibition of cAMP-dependent protein kinase by inhibitory peptides corresponding to the active portion of the heat-stable inhibitor protein. *J Biol Chem* **264**, 8802-8810 (1989).

32. Ishida A, Kameshita I, Okuno S, Kitani T, Fujisawa H. A novel highly specific and potent inhibitor of calmodulin-dependent protein kinase II. *Biochem Biophys Res Commun* **212**, 806-812 (1995).

33. Tokumitsu H, Chijiwa T, Hagiwara M, Mizutani A, Terasawa M, Hidaka H. KN-62, 1-[N,O-bis(5-isoquinolinesulfonyl)-N-methyl-L-tyrosyl]-4-phenylpiperazine, a specific inhibitor of Ca2+/calmodulin-dependent protein kinase II. *J Biol Chem* **265**, 4315-4320 (1990).

34. Anderson ME*, et al.* KN-93, an inhibitor of multifunctional Ca++/calmodulin-dependent protein kinase, decreases early afterdepolarizations in rabbit heart. *J Pharmacol Exp Ther* **287**, 996-1006 (1998).

35. Steigedal TS, Bruland T, Misund K, Thommesen L, Laegreid A. Inducible cAMP early repressor suppresses gastrin-mediated activation of cyclin D1 and c-fos gene expression. *American journal of physiology Gastrointestinal and liver physiology* **292**, G1062-1069 (2007).

36. Green SA, Holt BD, Liggett SB. Beta 1- and beta 2-adrenergic receptors display subtype-selective coupling to Gs. *Mol Pharmacol* **41**, 889-893 (1992).

37. Zheng M, Han QD, Xiao RP. Distinct beta-adrenergic receptor subtype signaling in the heart and their pathophysiological relevance. *Sheng Li Xue Bao* **56**, 1-15 (2004).

38. Berlot CH, Bourne HR. Identification of effector-activating residues of Gs alpha. *Cell* **68**, 911-922 (1992).

39. Gardner LA, Delos Santos NM, Matta SG, Whitt MA, Bahouth SW. Role of the cyclic AMP-dependent protein kinase in homologous resensitization of the beta1-adrenergic receptor. *J Biol Chem* **279**, 21135-21143 (2004).

40. Baillie GS*, et al.* beta-Arrestin-mediated PDE4 cAMP phosphodiesterase recruitment regulates beta-adrenoceptor switching from Gs to Gi. *Proc Natl Acad Sci U S A* **100**, 940-945 (2003).

41. Noma T*, et al.* Beta-arrestin-mediated beta1-adrenergic receptor transactivation of the EGFR confers cardioprotection. *J Clin Invest* **117**, 2445-2458 (2007).

42. Reiter E, Lefkowitz RJ. GRKs and beta-arrestins: roles in receptor silencing, trafficking and signaling. *Trends Endocrinol Metab* **17**, 159-165 (2006).

43. Moore CA, Milano SK, Benovic JL. Regulation of receptor trafficking by GRKs and arrestins. *Annu Rev Physiol* **69**, 451-482 (2007).

44. Ding B*, et al.* A positive feedback loop of phosphodiesterase 3 (PDE3) and inducible cAMP early repressor (ICER) leads to cardiomyocyte apoptosis. *Proc Natl Acad Sci U S A* **102**, 14771-14776 (2005).

45. Yehia G, Schlotter F, Razavi R, Alessandrini A, Molina CA. Mitogen-activated protein kinase phosphorylates and targets inducible cAMP early repressor to ubiquitin-mediated destruction. *J Biol Chem* **276**, 35272-35279 (2001).

46. Xiang YK. Compartmentalization of beta-adrenergic signals in cardiomyocytes. *Circ Res* **109**, 231-244 (2011).

47. Ding B*, et al.* Functional role of phosphodiesterase 3 in cardiomyocyte apoptosis: implication in heart failure. *Circulation* **111**, 2469-2476 (2005).

48. Yao G, Tan C, West M, Nevins JR, You L. Origin of bistability underlying mammalian cell cycle entry. *Mol Syst Biol* **7**, 485 (2011).

49. Xing J, Ginty DD, Greenberg ME. Coupling of the RAS-MAPK pathway to gene activation by RSK2, a growth factor-regulated CREB kinase. *Science* **273**, 959-963 (1996).

50. De Cesare D, Jacquot S, Hanauer A, Sassone-Corsi P. Rsk-2 activity is necessary for epidermal growth factor-induced phosphorylation of CREB protein and transcription of c-fos gene. *Proc Natl Acad Sci U S A* **95**, 12202-12207 (1998).

51. Grimm M, Brown JH. Beta-adrenergic receptor signaling in the heart: role of CaMKII. *J Mol Cell Cardiol* **48**, 322-330 (2010).

52. Qian H. Phosphorylation energy hypothesis: open chemical systems and their biological functions. *Annu Rev Phys Chem* **58**, 113-142 (2007).

53. Saucerman JJ, Bers DM. Calmodulin mediates differential sensitivity of CaMKII and calcineurin to local Ca2+ in cardiac myocytes. *Biophys J* **95**, 4597-4612 (2008).
